# Mucosal immunity and vaccination strategies: current insights and future perspectives

**DOI:** 10.1186/s43556-025-00301-7

**Published:** 2025-08-20

**Authors:** Zhihao Zhang, Weiqi Hong, Yu Zhang, Xin Li, Haiying Que, Xiawei Wei

**Affiliations:** 1 State Key Laboratory of Biotherapy and Cancer Center, West China Hospital, Sichuan University, Chengdu, Sichuan China; 2 West China School of Pharmacy, Sichuan University, Chengdu, Sichuan China

**Keywords:** Mucosal immunity, Vaccine development, Mucosal technology platform, Adjuvants, Vaccine delivery

## Abstract

The mucosal immune system represents a critical defense mechanism, safeguarding the body from an array of external pathogens. As the body’s first line of immune protection, it plays an essential role in initiating both innate and adaptive immune responses. Through intricate networks of immune cells and complex molecular pathways, mucosal immunity orchestrates a robust defense not only at the local level but also activates systemic immune responses to ensure comprehensive protection. Consequently, the mucosal immune system has garnered immense interest in the field of vaccine development, given its potential to foster durable and effective immunization. Despite the profound promise of mucosal immunity, the development of mucosal vaccines faces significant challenges, particularly with existing technological platforms that primarily rely on live attenuated or inactivated vaccines. However, emerging innovative platforms, including subunit vaccines, viral vector vaccines, and the groundbreaking application of mRNA vaccines, are offering new perspectives, vastly improving the scope and efficacy of mucosal immunization. As mucosal immunity research continues to evolve, rapid advancements in biotechnology and immunology provide promising strategies to enhance immune responses and overcome inherent limitations. This review delves into the latest progress in oral, nasal, and other forms of mucosal vaccines, analyzing the intricate relationship between mucosal immune characteristics and vaccine design. Emphasis is placed on the pivotal role of advanced adjuvants and delivery systems in maximizing vaccine efficacy. This review addresses current challenges, highlights future research opportunities, and aims to provide a comprehensive framework for advancing the field of mucosal immunity and vaccine development.

## Introduction

Mucosal immunity refers to the immune responses that occur at the surfaces of various mucosal tissues in the body. It is a vital component of the body’s defense system, encompassing immune responses in regions such as the gastrointestinal, respiratory, and urogenital tracts. This immune system is composed of innate immune components, immune cells (such as innate lymphoid cells [ILCs], tissue-resident memory T cells), and antibodies, primarily immunoglobulin A (IgA). These elements work synergistically to maintain the integrity of the mucosal barrier and effectively defend against pathogen invasion [1, 2]. Mucosal immunity not only triggers immune responses in local and distant mucosal sites but also can generate systemic reactions to suppress further invasion by primary pathogens [3, 4].

With the outbreak of the COVID-19 pandemic, the significance of mucosal immunity has been further emphasized, driven by a deeper understanding of its underlying mechanisms. In addition to the respiratory mucosa, mucosal immunity in the gastrointestinal and urogenital tracts is also facing challenges from emerging infections [5, 6]. Mucus, peristalsis, gastric acid, bile, and antimicrobial peptides constitute the innate mucosal immune strategies, while adaptive mucosal immune responses include antigen-specific antibodies and cell-mediated reactions [4, 7]. Among the factors related to mucosal immunity, inducing antigen-specific IgA is a key consideration, as IgA is the predominant antibody in many mucosal sites. IgA molecules form polymers by binding to pathogens, thereby enhancing their ability to clear the pathogens. This mechanism helps improve the efficiency of mucosal immune responses and plays a crucial role in preventing pathogens from invading host tissues. Recently, the importance of dimeric IgA in neutralizing respiratory viruses, including SARS-CoV-2, has been highlighted [8]. Highly active IgA protects against intestinal pathogens through agglutination and a recently described process known as "chain growth" [9]. In addition to IgA, it is important to highlight that tissue-resident T cells are a subset of crucial mucosal effector cells with memory characteristics [10], which remain in non-lymphoid tissues for extended periods. These cells are found in almost all tissues, and upon encountering a pathogen for the second time, they quickly exert effector functions, thereby limiting the progression of the disease [11]. When mucosal surfaces are directly immunized, rather than through systemic pathways, both approaches can trigger a more robust and lasting immune response [12, 13].

The ideal mucosal vaccine should be capable of eliciting a sustained and effective local mucosal immune as well as a systemic immune response, thereby strengthening the body's overall immunity. Over the past decade, global scientific and pharmaceutical communities have focused on developing vaccines against various pathogens, especially respiratory and gastrointestinal pathogens. In designing mucosal vaccines or evaluating their necessity, it is crucial to recognize that the key to controlling pandemics lies in both reducing disease severity and effectively interrupting virus transmission [14]. While most recent advancements have been in injectable vaccines, these traditional vaccines offer a certain degree of preventive efficacy. However, they often fall short in preventing viral transmission, as they may not significantly reduce viral shedding at the site of infection/entry [7]. Mucosal vaccines, which elicit robust immune responses at mucosal surfaces such as the respiratory and gastrointestinal tracts, provide a promising approach to preventing pathogen transmission [2, 13, 15]. Furthermore, mucosal vaccines are typically administered orally or nasally, making vaccination more convenient and improving patient acceptance, especially among vulnerable populations such as children and the elderly, while avoiding the discomfort associated with injections [5, 13, 16].

With the growing global attention on pathogens adhering to mucosal surfaces, respiratory pathogens remain one of the leading causes of death worldwide, with lower respiratory tract infections ranking as the fourth leading cause of death globally. Approximately 2.4 million people die annually from lower respiratory tract infections, with pathogens such as Streptococcus pneumoniae, Respiratory Syncytial Virus (RSV), Haemophilus influenzae type B, and influenza viruses, particularly posing a high risk of mortality for children under five and the elderly [17]. Although vaccines have been developed for pathogens such as Streptococcus pneumoniae, Mycobacterium tuberculosis, Bordetella pertussis, influenza virus, typhoid, hepatitis B, and Human Papillomavirus (HPV), they are all mainly administered via injection. However, vaccine development against these pathogens is increasingly focused on mucosal vaccines to enhance local immune responses [18–23].

Existing mucosal vaccines rely on the use of attenuated or inactivated pathogens, limiting their applicability in responding to emerging pathogens. To date, the FDA has approved only nine mucosal vaccines for human use, eight of which are oral vaccines (Table 1), with just one being administered intranasally (FluMist by MedImmune/Sanofi Pasteur). [12, 24]. Additionally, vaccine stability, optimization of delivery systems, and persistence of immune responses remain critical challenges in mucosal vaccine development. A deeper exploration of mucosal immune mechanisms and the functions of associated immune cells can provide essential theoretical foundations for vaccine design, accelerating their adoption in global public health. This review systematically analyzes the mechanisms underlying mucosal immunity and current technological platforms for mucosal vaccines, and discusses future directions in developing next-generation mucosal vaccines. Ultimately, this work aims to enhance public understanding of mucosal immunity and to highlight its potential impact on global health.

**Table 1:** FDA approved mucosal vaccine

Infection	Vaccine	Composition	Technological platform	Mucosal Route	Approval Year
Vibrio cholerae	Dukoral	heat and formaldehydeinactivated O1 serogroups (Inaba + Ogawa) + CTB	Inactivated	Oral-aqueous	1997
	Euvichol, Shanchol	heat and formaldehydeinactivated O1 serogroups (Inaba + Ogawa) + 0139	Inactivated	Oral-aqueous	2011
	Vaxchora	Live attenuated 01 serogroup (Inaba): ctxA attenuation	Live attenuated	Oral-aqueous	2015
Poliovirus	Biopolio (bOPV)	culture passage attenuated polioviruses 1 and 3 serotypes (5′ non-coding region attenuation)	Live attenuated	Oral-aqueous	1961
	mOPV and tOPV	culture passage attenuated polioviruses 1, 2, and 3 serotypes (5′ non-coding region attenuation)	Live attenuated	Oral-aqueous	1961
Influenza A and influenza B viruses	FluMist/Fluenz	quadrivalent antigens from circulating strains incorporated into live attenuated, cold-adapted donor influenza vector	Live attenuated/reassortant	Nasal-spray	2003
Salmonella typhimurium	Typhi Vivotif	Live attenuated Ty21a strain Mutagenesis in LPS synthesis and Vi polysaccharide genes	Live attenuated/reassortant	Oral-capsule	2013
Rotavirus	Rotateq	pentavalent-five human–bovine reassortant rotaviruses (expression of G1, G2, G3, G4, G5 with P7 and G6 with P1A)	Live reassortant	Oral-aqueous	2006
	Rotarix	monovalent-culture passage attenuated (G1 with P1A expression)	Live attenuated	Oral-aqueous	2008
Febrile acute respiratory diseases	Adenovirus Type 4 and 7 Vaccine (Barr Labs) (approved only in new military recruits in the US)	Live-attenuated adenovirus type 4 and type 7	Live attenuated adenovirus vaccine	Oral–Enteric-coated tablet	2011

Data from website of Vaccines Licensed for Use in the United States (https://www.fda.gov/vaccines-blood-biologics/vaccines/vaccines-licensed-use-united-states)

## Mucosal immunity and its effector cells and molecules

To understand how mucosal vaccines can be optimized, it is crucial to first examine the cellular and molecular components that constitute mucosal immunity. This section systematically delineates the core effector cells and critical molecular immune mechanisms of the mucosal immune system, including the physical and chemical barriers serving as the first line of defense, the trained immunity mechanism enhancing immune defenses upon repeated pathogen exposure, key processes in adaptive immune responses, and the functional characteristics of essential effector antibodies (such as IgA) and tissue-resident memory lymphocytes (T_RM_). A comprehensive understanding of these fundamental mechanisms deepens our insight into how mucosal immunity effectively counters pathogen invasion and provides a solid theoretical foundation and conceptual framework for designing and optimizing mucosal vaccines capable of eliciting durable protection.

### First line of defense: physical and chemical barrier and the training of mucosal innate immunity

The mucosal surface area is approximately 200 times greater than that of the skin. The mucosa serves as the first line of defense of the host immune system against pathogen and allergen invasion and is distributed across various essential organs, and is categorized into two distinct types. Type I mucosa is predominantly found in the respiratory tract and most of the gastrointestinal tract, while type II mucosa encompasses the oral cavity and the urogenital tract [1, 12, 25]. The innate immune system consists of physical, chemical, and biological factors located at epithelial, subepithelial, and epithelial surface levels. Physical factors include the epithelial tight junctions (such as tight junction proteins and adhesion proteins) encased in secreted mucus, forming a physical barrier. Additionally, ciliated cells in the respiratory epithelium play a pivotal role in clearing mucus by expelling pathogens and particles from the mucosal surfaces. Similarly, the peristalsis of the intestinal mucosa, along with the continuous renewal and repair of epithelial cells in the gastrointestinal tract, significantly reduces the risk of pathogen invasion [26, 27].

Biochemical factors encompass the microbiota present in the lumen and antimicrobial peptides, which collectively act as biological and biochemical barriers. Healthy microbiota can inhibit pathogen survival through competitive exclusion, secreting antimicrobial substances such as lactic acid, acetic acid, and antimicrobial peptides, among others. Moreover, normal microbiota promotes the development and function of the immune system, activates immune responses, and helps maintain the stability of the mucosal surfaces. Mucus contains various antimicrobial components, such as antimicrobial peptides, lysozyme, and lactoferrin. These substances exhibit broad-spectrum antimicrobial activity and are capable of directly inhibiting or eliminating pathogenic microorganisms. Specifically, antimicrobial peptides (AMPs) are a class of chemical factors produced by epithelial and immune cells. This group includes human β-defensins (HBDs) and secretory leukocyte protease inhibitors (SLPIs), which are upregulated during infections and exhibit potent antimicrobial properties [28–31]. Antimicrobial peptides (AMPs) activate inflammatory responses and regulate immune reactions by recognizing pathogen-associated molecular patterns (PAMPs) and damage-associated molecular patterns (DAMPs) [11, 32].

Furthermore, epithelial cells in the gastrointestinal tract and other mucosal sites secrete digestive enzymes and antibodies (such as IgA), which play a significant role in immune defense [12, 25, 33, 34]. When pathogens and microbial molecules breach the body's two natural barriers—physical and biochemical barriers—innate immune cells present in the subepithelial tissues, including macrophages, mast cells, natural killer (NK) cells, and innate lymphoid cells, respond rapidly to initiate a defensive reaction [11]. These immune cells recognize and combat the invading pathogens through phagocytosis or Pattern Recognition Receptors (PRRs). For instance, macrophages can recognize pathogen-associated molecular patterns (PAMPs) through receptors such as TLRs, NOD-like receptors (NLRs), and RIG-I-like receptors (RLRs) on their surface. Upon binding of these receptors with pathogenic material, macrophages can detect and respond to pathogen invasion [35]. Subsequently, macrophages ingest pathogens via phagocytosis and degrade them through enzymes within lysosomes. Moreover, macrophages activate associated signaling pathways and secrete a range of cytokines, such as tumor necrosis factor-alpha (TNF-α), interleukin-1 (IL-1), and interferon-gamma (IFN-γ). These cytokines not only directly kill pathogens but also recruit additional immune cells, further enhancing the local inflammatory response and immune defense mechanisms [36]. Additionally, mast cells can recognize antigens or microbial pathogens through their high-affinity immunoglobulin E (IgE) receptors (FcεRI). When pathogens or allergens bind to the IgE on mast cells, the cells undergo degranulation, releasing a series of bioactive substances, including histamine, leukotrienes, and prostaglandins. This degranulation process triggers local vasodilation and increased vascular permeability, thereby facilitating the infiltration of immune cells and enhancing pathogen clearance [37].

### Trained immunity

Trained immunity refers to the enhanced immune response capacity of the innate immune system following repeated exposure to pathogens, mediated by adaptive changes [38]. This phenomenon broadly impacts the entire mucosal immune system, including the respiratory, gastrointestinal, and urogenital tracts, as well as other local mucosal barriers, contributing to improved immune defense functions [39]. Trained immunity is characterized by heightened innate immune responsiveness to subsequent infections caused by unrelated pathogens, thus providing broad protection against heterologous infections [40]. Research has demonstrated that trained immunity differs significantly from immune tolerance. While immune cells in trained immunity undergo a programmed"activation"process that enhances their effector functions, tolerance involves programmed alterations that suppress immune cell activity. Central to the development of trained immunity are coordinated metabolic and epigenetic mechanisms. During the initial immune challenge, PRRs engage and activate several metabolic pathways, particularly glycolysis, the tricarboxylic acid (TCA) cycle, and fatty acid metabolism. The products of these metabolic pathways induce epigenetic changes in chromatin, influencing gene regions critical to innate immune responses [41].

Furthermore, trained immunity is antigen-independent and can persist for periods ranging from six months to five years [18, 40, 42]. It can be gradually activated and strengthened through vaccination (e.g., Bacillus Calmette-Guérin [BCG], oral polio vaccine [OPV], smallpox, measles, mumps, and rubella [MMR] vaccines), β-glucan components, or microbial infections (e.g., Candida albicans, hepatitis B virus) [42–46]. Notably, trained immunity can also be induced by non-microbial sources, as documented in other studies, though these details are beyond the scope of the current discussion [18]. Among these, the role of BCG-induced trained immunity is well-established. Upon entering the body, BCG activates PRRs in the innate immune system, particularly those from the Toll-like receptor (TLR) family, significantly inducing the production of pro-inflammatory cytokines such as IL-6, IL-1β, and tumor necrosis factor (TNF) [47]. These pro-inflammatory cytokines then stimulate antigen epitopes, promoting subsequent adaptive immune responses [48, 49]. As a result, multiple studies have reported that BCG successfully induces long-term trained immunity against various pathogens, including COVID-19, thereby enhancing mucosal defense and providing protection against respiratory infections [50–52]. In summary, trained immunity provides an effective immune defense mechanism by enhancing immune memory and local defense capabilities of the mucosal immune system. With a more comprehensive understanding of the underlying mechanisms of trained immunity, future vaccine development and immunological interventions can be more precisely tailored, better harnessing this mechanism to offer robust immune protection for the prevention and treatment of infectious diseases. With trained immunity enhancing innate immune responses, the adaptive immune system plays a crucial role in long-term defense.

### Adaptive immune response mechanism of mucosal immunity

Mucosa-associated lymphoid tissue (MALT) refers to lymphoid tissues located near the mucosal surfaces of the body, such as those in the respiratory, gastrointestinal, and urogenital tracts. It is functionally divided into inductive sites and effector sites [53]. The inductive sites are responsible for activating antigen-specific T and B cell responses, while the effector sites, such as the lamina propria and epithelium, execute the actual defense functions of immune responses [54]. The coordinated activity of this immune network is essential for the efficacy of mucosal immunity and the maintenance of overall health. The characteristics of the inductive sites vary between species and across different mucosal tissues [55, 56]. Mucosal immune induction sites, composed of mucosa-associated lymphoid tissue (MALT), include gut-associated lymphoid tissue (GALT) and nasopharynx-associated lymphoid tissue (NALT). GALT, located in the gastrointestinal tract (e.g., Peyer’s patches, appendix, mesenteric lymph nodes), initiates intestinal immune responses, while NALT, found in the nasopharynx (e.g., palatine and pharyngeal tonsils), is essential for defense against respiratory pathogens. These sites are primarily composed of dendritic cells, macrophages, innate lymphoid cells, mucosa-associated invariant T cells, intraepithelial T cells, regulatory T cells (Treg), plasma cells secreting IgA, as well as memory B and T cells that migrate to effector sites to initiate immune responses [57].

**Fig. 1:**
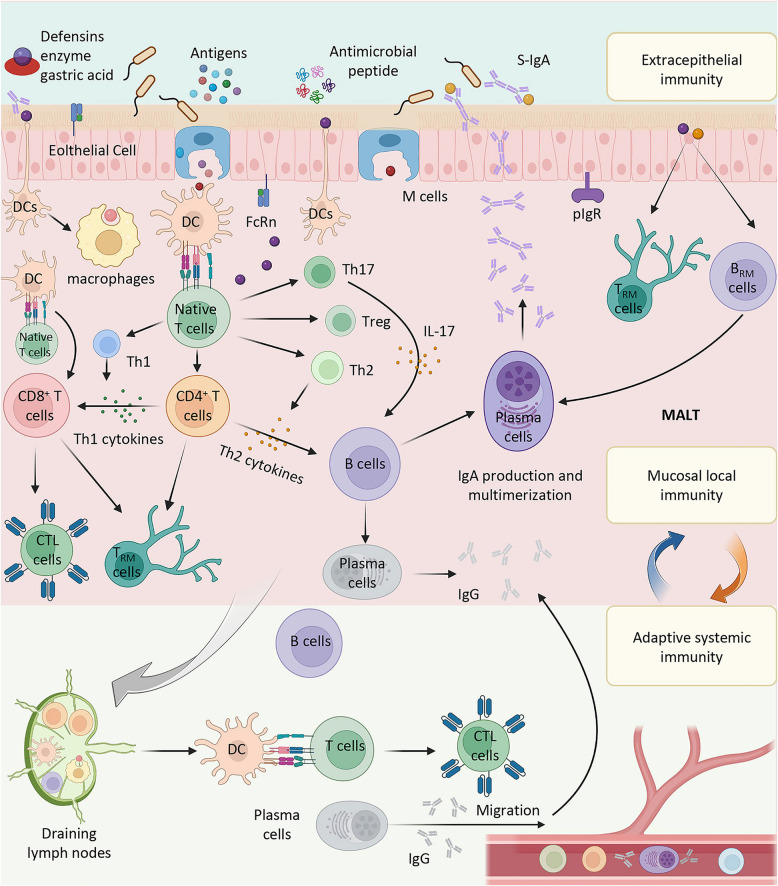
The Mechanism of Mucosal Immunity. The mucosal immune system comprises key cells and molecular components involved in mucosal immune responses. At the inductive site, antigens are captured by M cells and presented to dendritic cells (DCs), which then activate naive T cells, including CD8^+^ cytotoxic T lymphocytes (CTLs), Th1, Th2, Th17, and regulatory T cells (Tregs). These activated T cells, along with B cells, contribute to local immune responses at the effector site in mucosa-associated lymphoid tissues (MALT), leading to the production of secretory IgA (S-IgA) and antimicrobial peptides, such as defensins. Plasma cells within the mucosa play a crucial role in the production and multimerization of IgA. Additionally, systemic immune responses are activated, with plasma cells migrating to the effector site to produce IgG, which is transported across epithelial cells via the pIgR receptor. The role of memory T cells (T_RM_ cells) and the interactions between innate and adaptive immunity are also highlighted, emphasizing the complexity of mucosal immune regulation in defending against pathogens

The initiation of adaptive mucosal immune responses begins with antigen presentation (Fig. 1). M cells, specialized epithelial cells, play a pivotal role in this process. Located in the epithelial layer, M cells efficiently absorb antigens from the external environment and transport them to underlying immune cells, such as dendritic cells (DCs) and macrophages. These cells, as primary antigen-presenting cells, recognize and ingest pathogens. After activation and maturation, they present antigens to naïve T cells. Due to the unique properties of M cells, which do not secrete mucus or glycocalyx and possess high endocytic activity, they can efficiently capture and transport pathogens. Concurrently, epithelial cells secrete pro-inflammatory cytokines, further stimulating the immune cells, thereby enhancing the immune response [7].

Upon activation, T cells undergo clonal expansion and differentiate into various subsets, including Th1, Th2, Th17, and Treg cells [58]. Each subset secretes distinct cytokines that regulate immune responses. For example, Th17 cells secrete interleukin-17 (IL-17), which upregulates the expression of polymeric immunoglobulin receptors (pIgR) on mucosal epithelial cells, thereby promoting the production of secretory IgA (S-IgA). This process is crucial for enhancing vaccine-induced protective mucosal immunity [1]. T cells also activate B cells through the regulation of transcription factors and the secretion of lineage-specific cytokines. Upon activation, B cells differentiate into plasma cells and begin producing immunoglobulins, such as IgA and IgG. Among these, IgA plays a central role in mucosal immunity. Mucosal IgA binds to pIgR on mucosal epithelial cells, facilitating the transport of antibodies to the mucosal surface, thereby forming a mucosal barrier that effectively prevents pathogen adhesion and invasion [59].

On the other hand, most extra-intestinal immune responses can also induce a systemic IgG response through the migration of IgG-producing B cells, activating DCs, and facilitating the migration from the mucosa to the bone marrow, lymph nodes, and spleen [60]. Cytotoxic T lymphocyte (CTL) responses can also be induced at mucosal sites to clear mucosal microbes [58]. Although mucosal immune responses are typically compartmentalized, there is a crosstalk between different mucosal sites. As a result, vaccines administered at a single mucosal site can promote immune responses at distant mucosal locations. Understanding the nature of signals that regulate this homing process in the human environment is crucial for designing novel mucosal vaccines. These vaccines could potentially target mucosal sites far from the site of vaccination [61].

### IgA and other mucosal antibodies

The production of IgA plays a crucial role in mucosal immunity, particularly in defending against pathogen invasion. IgA class-switch recombination occurs via two mechanisms: T cell-dependent (TD) and T cell-independent (TI). The TD pathway requires CD40/CD40L signaling to induce the generation of high-affinity IgA antibodies [62]. In contrast, the TI pathway is mediated by innate lymphoid cells (ILCs) and plasmacytoid dendritic cells (pDCs), which secrete B cell-activating factor (BAFF) and a proliferation-inducing ligand (APRIL) to promote IgA responses against commensal microbiota [63, 64]. Human B cells produce two IgA subclasses, IgA1 and IgA2, which share similar receptor-binding affinities but differ in structural configuration [65]. IgA1 is predominantly distributed in the bloodstream and certain mucosal tissues, while IgA2 is enriched in microbe-dense environments such as the distal intestine and the urogenital tract [12, 66].

Secretory IgA (S-IgA) in mucosal regions and IgG in the circulatory system are the primary effector molecules at mucosal sites. In the upper respiratory tract and other mucosal surfaces, S-IgA is the dominant immunoglobulin, typically present at levels approximately three times higher than IgG, playing a pivotal role in preventing infections at mucosal sites [38]. Unlike the respiratory mucosa, S-IgA in the intestine is primarily produced by GALT, specifically Peyer's patches (PP). PP serves as the main precursor for IgA-producing plasma cells, secreting approximately 3 g of S-IgA into the intestinal lumen of an adult per day [67]. In effector sites such as the lamina propria, IgA secreted by plasma cells binds to the polymeric immunoglobulin receptor (pIgR) on the basolateral side of epithelial cells for transport to the mucosal surface. During this transport process, the extracellular part of pIgR is cleaved, forming the secretory component (SC), which subsequently constitutes secretory IgA (S-IgA) [68]. S-IgA predominantly exists as a dimer in mucosal secretions and exhibits higher affinity and neutralizing capacity than both IgG and monomeric IgA. It effectively prevents pathogen adhesion, colonization, and invasion at mucosal surfaces, thereby preserving the integrity of the mucosal barrier [69]. The multiple antigen-binding sites of S-IgA may contribute to its efficient protective capabilities [8, 9, 38, 70, 71]. Furthermore, S-IgA has inherent proteolytic resistance, ensuring its stability in mucosal secretions rich in proteases [4].

Clinical studies indicate that in respiratory viral infections such as influenza and SARS-CoV-2, the specific S-IgA levels rise rapidly between 7 and 15 days post-infection and remain stable over the following months (typically 3 to 9 months) [72, 73]. Research also shows that antigen-specific S-IgA is closely associated with the prevention of respiratory viral infections [73–75]. For example, a study by Sho et al. highlighted that the anti-spike protein (S protein) S-IgA response in SARS-CoV-2-infected individuals significantly reduced the risk of viral transmission and viral shedding in the respiratory tract, indicating a strong correlation between early IgA response and viral clearance [76]. Based on these findings, the role of S-IgA in mucosal immunity has been further clarified. It not only blocks pathogen invasion but also promotes local immune defense by maintaining the homeostasis of the mucosal microenvironment. Therefore, developing mucosal vaccines that effectively induce antigen-specific S-IgA responses has become a key strategy for preventing mucosal infectious diseases, including respiratory viral infections.

### Tissue resident memory lymphocytes

In addition to IgA serving as a key humoral effector molecule in the mucosal immune system, certain memory lymphocytes also play critical roles in mucosal immunity. Tissue-resident memory B cells (B_RM_ cells) act as a long-term source of local IgA responses; they reside and persist within mucosa-associated lymphoid tissues (MALT) or local barrier tissues and, upon re-exposure to antigen, rapidly differentiate into IgA-secreting plasma cells, enabling a swift and antigen-specific antibody response[77]. Moreover, IgA antibodies and T_RM_ cells represent the key humoral and cellular effector mechanisms, respectively, within the mucosal immune system, and offer highly complementary functional value in vaccine design. S-IgA is primarily localized at mucosal surfaces, where it effectively prevents pathogen adhesion and invasion through neutralization and polymeric exclusion mechanisms [77]. In contrast, T_RM_ cells reside in subepithelial tissues and are capable of rapidly initiating cytotoxic or helper immune responses upon pathogen breach of the initial barrier, thereby eliminating infected cells [78]. Together, these two components form a dual-layered local defense strategy of “blockade and clearance” at mucosal sites.

In mucosal vaccine strategies, a key challenge lies in coordinating the induction of both IgA secretion and T_RM_ cell establishment through the optimization of adjuvant combinations, vaccine platforms, and delivery routes [12]. For example, intranasal administration of live-attenuated viral vaccines effectively stimulates T_RM_ responses [24], while oral adjuvanted subunit vaccines are more suitable for inducing durable IgA production [79]. Therefore, harnessing the synergistic mechanisms of IgA and T_RM_ cells is fundamental to achieving broad, potent, and long-lasting protection in mucosal vaccine design.

Tissue-resident memory cells (T_RM_), comprising both T and B lymphocytes, persist stably within non-lymphoid barrier tissues such as the skin, lungs, and intestines, as well as non-barrier tissues including the brain and liver. These cells function as immune sentinels, capable of rapidly initiating localized immune responses upon secondary pathogen invasion at their respective tissue sites, thereby mediating swift and antigen-specific recall immunity [80].

#### Tissue-resident memory T cells

T_RM_ cells are able to persist in local tissues, particularly at epithelial barriers such as the skin [81], lungs, and intestines. Their tissue retention is enhanced by the expression of CD69 and CD103, which regulate sphingosine-1-phosphate receptor 1 (S1PR1) and interact with epithelial E-cadherin, thereby promoting their residency within the tissue microenvironment [82, 83]. Studies show that T_RM_ cells express various tissue-homing chemokine receptors, which help them localize to specific tissue sites. CXCR6, the chemokine receptor for CXCL16, is highly expressed on human T_RM_ cells in the lungs, liver, and lymphoid tissues [11]. Mouse studies indicate that CXCR6 is essential for recruiting CD8^+^ T_RM_ cells to mucosal epithelia [84]. CXCR3, a receptor for chemokines CXCL9, CXCL10, and CXCL11, is also expressed on a portion of lung T_RM_ cells [85]. The expression of these chemokine receptors enables T_RM_ cells to persist long-term in local tissues and rapidly exert effector functions upon encountering the same pathogen, limiting disease progression. Studies show that the number of T_RM_ cells in the airways is negatively correlated with the severity of respiratory viral infections. In an experiment involving respiratory syncytial virus (RSV) infection, volunteers with higher levels of T_RM_ cells in their bronchoalveolar lavage fluid exhibited milder symptoms [86]. Additionally, CD8^+^ T_RM_ cells in the nasal cavity effectively prevent the spread of the virus to the lungs, significantly reducing the severity of pulmonary disease [87]. These findings further validate the crucial role of T_RM_ cells in protecting the respiratory tract from pathogen invasion and highlight their active defensive role in antiviral immunity. In addition, Effective tumor immune surveillance and elimination depend on tumor-specific CD8^+^ T cells [88], which offers another important connection to mucosal vaccine efficacy.

CD4^+^ tissue-resident memory T cells (CD4^+^ T_RM_ cells) have a broader distribution in tissues compared to CD8^+^ T_RM_ cells and play a key role in supporting the establishment of immune responses of tissue-resident memory B cells and CD8^+^ T_RM_ cells [89]. In murine lung models, CD4⁺ T_RM_ cells enhance local immune responses by secreting interferon-gamma (IFN-γ), thereby promoting the formation of both CD8⁺ T_RM_ cells and memory B cells, which act synergistically in antiviral defense [90, 91]. Recent studies have shown that human duodenal CD4^+^ T cell compartments are rich in multifunctional TH1 cell populations that survive for at least one year. Intranasal vaccine studies have shown that successful induction of CD4^+^ T_RM_ cells in animals can prevent pneumococcal colonization, influenza attacks, and SARS-CoV infection [53, 92, 93].

Although T_RM_ cells, particularly CD8^+^ in the lungs, are crucial for defending against respiratory viral infections, these cells typically have a relatively short lifespan, which may impair responses to subsequent infections [94]. One possible explanation is that lung T_RM_ cells may migrate to the mediastinal lymph nodes via a process known as "retrograde migration". Some solutions have shown potential to promote the long-term maintenance of lung T_RM_ cells, including using systemic booster vaccines to increase circulating effector memory T cells or using viral vector vaccines to extend antigen retention time in the lungs [95].

#### Tissue-resident memory B cells

Memory B cells (B_RM_ cells) rapidly differentiate into antibody-secreting cells, such as plasma cells, upon antigen re-exposure, producing antibodies to prevent reinfection by pathogens. Similar to T_RM_ cells, B_RM_ cells reside in mucosal tissues and enhance local secondary immune responses through their interaction with antigen-presenting cells and T cells [96, 97]. For example, in the case of influenza virus reinfection, local immunity can quickly generate inducible bronchus-associated lymphoid tissue (iBALT), supporting the maturation and selection of B cells, leading to the generation of B_RM_ cells and the establishment of resident memory T follicular helper cells (TFH) [98]. CXCR3^+^ and CCR6^+^ virus-specific B cells are generated after influenza and SARS-CoV-2 infections. CXCR3^+^ B_RM_ cells rapidly respond during reinfection in the lungs by migrating to the infection site through chemotaxis, providing strong local immune protection [99]. Furthermore, adoptive transfer studies have shown that compared to memory B cells isolated from the spleen, lung B_RM_ cells reduce viral titers in the lower respiratory tract [100]. In addition to antigen-specific B_RM_ cells, bystander B_RM_ populations provide secondary functions by retaining and presenting exogenous antigens in the form of immune complexes [80, 101, 102]. Future research needs to further explore the functions and formation mechanisms of tissue-resident cells. Based on these mechanisms, better strategies should be developed to induce more tissue-resident cells through mucosal vaccines, thereby enhancing the efficacy of the vaccines.

## Mucosa-associated lymphoid tissue

Following the discussion on key effector cells and molecular mechanisms of mucosal immunity, this section further explores the tissue-specific immune architecture and functional characteristics of mucosa-associated lymphoid tissues (MALT) across different mucosal sites, including the respiratory, gastrointestinal, and urogenital tracts. These specialized immune tissues not only play critical roles in local pathogen defense but also offer unique targeting characteristics for vaccine antigen delivery and immune induction. By elucidating the immunological functions specific to these tissues, this section provides a robust histological and immunological theoretical basis for developing customized vaccine delivery strategies tailored to diverse mucosal sites.

### Respiratory mucosal immunity

The epithelial cells of the respiratory tract are regarded as the primary defense barrier against invasive viral infections [103]. The airway mucosal surface is coated with various fluids, including mucus, antimicrobial peptides (AMPs), and enzymes, which play essential roles in innate immunity by capturing and eliminating invading pathogens and particulate matter. Notably, the respiratory mucosa is covered by a thick mucus layer, which serves as an additional protective measure. Mucus is primarily composed of O-glycosylated mucins, which are classified into gel-forming mucins (such as MUC5AC and MUC5B) and transmembrane mucins (including MUC1, MUC4, and MUC16). Gel-forming mucins effectively facilitate pathogen clearance from the airways through ciliary movement. MUC1 is the most abundant, with MUC1 and MUC4 present in both the upper and lower respiratory tracts, whereas MUC16 is exclusively expressed in the lower respiratory tract. These mucins serve not only as a physical barrier but also as decoy receptors to trap pathogens. Additionally, the shedding of their extracellular domains promotes pathogen detachment from the epithelial surface into the lumen, thereby enhancing mucociliary clearance [104]. In addition to the mucus layer, several key immune cell populations contribute significantly to respiratory mucosal immunity.

In the context of innate immune responses, innate lymphoid cells (ILCs) play a pivotal role in immune regulation within the respiratory tract [105]. Unlike circulating lymphocytes, ILCs do not express antigen-specific T cell receptors (TCRs), which allows them to respond to pathogens in an antigen-independent manner by detecting signals and secreting cytokines [106]. Among them, ILC2s are rapidly activated by cytokines such as IL-33 and IL-25, leading to the induction of type 2 immune responses. They secrete immunoregulatory factors such as IL-10 and epithelial repair mediators to maintain airway homeostasis. Furthermore, through the upregulation of MHC class II expression via PD-1 (on Th2 cells) and ICOS (on ILC2s and regulatory T cells), ILC2s enhance epithelial barrier integrity and facilitate antigen presentation to CD4⁺ T cells [107, 108]. Furthermore, ILC3 cells have been shown to play a critical role in preventing secondary bacterial infections during influenza infection [109].

Alveolar macrophages represent a crucial subset of resident immune cells in the lungs. They not only participate in the early immune response by phagocytosing pathogens and clearing cellular debris but also contribute to immune homeostasis. These macrophages recruit additional immune cells by secreting pro-inflammatory cytokines such as IL-6 and TNF-α, while simultaneously producing anti-inflammatory factors such as IL-10 to modulate excessive immune responses, thus preventing immune dysregulation [110].

In the respiratory tract, mucosa-associated lymphoid tissue (MALT) includes the nasopharyngeal-associated lymphoid tissue (NALT) in the upper respiratory tract and the bronchus-associated lymphoid tissue (BALT) in the lower respiratory tract (LRT) [80]. NALT, which is similar to the Peyer’s patches (PP) in the small intestine, is often referred to as the Waldeyer’s ring of lymphoid tissues, primarily composed of the palatine tonsils and the pharyngeal tonsils. As an entry point for the upper respiratory tract, both the oral and nasal cavities play a dual role in preventing pathogen invasion and functioning as crucial immune organs. In the tonsils, lymphoid cells, mainly B cells, and myeloid cells are the most prominent immune cells. The surface epithelium follows the contours of the follicles and extends deep into the tonsils, forming invaginations that significantly increase the surface area by up to six times. At the deepest part of these invaginations, a lymphoepithelial symbiosis forms between the epithelium and the tonsillar parenchyma. In this region, antigen-presenting cells (such as M cells and dendritic cells), along with memory B cells, are abundantly expressed. Activated B cells differentiate into plasma cells and produce antibodies through somatic hypermutation to exert their effects [111, 112].

**Fig. 2:**
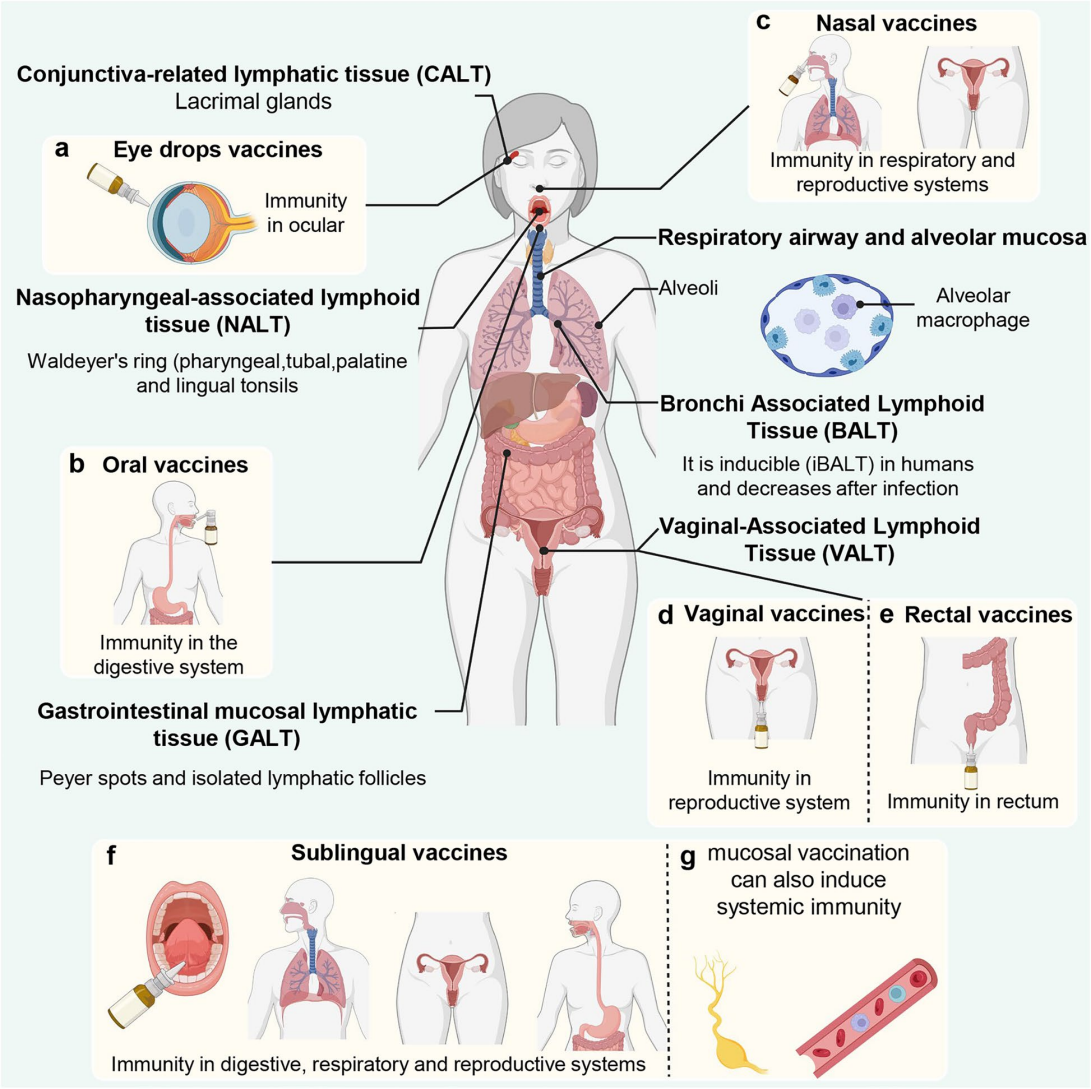
Human mucosal-associated lymphoid tissues and mucosal vaccination routes. The key mucosal lymphoid tissues in the human body include those associated with the conjunctiva (CALT), nasopharynx (NALT), respiratory tract (BALT), gastrointestinal tract (GALT), and vagina (VALT). Additionally, various mucosal vaccination routes, such as nasal, oral, ocular (eye drops), vaginal, rectal, and sublingual routes, can stimulate local immune responses in the corresponding mucosal tissues. Each route has the potential to induce both local and systemic immune responses. This highlights the importance of targeting mucosal immunity in different systems, such as the respiratory, digestive, reproductive, and ocular systems, to enhance protection against pathogens

In the context of respiratory mucosal immunity, different vaccination routes can induce variations in the potency and duration of immune responses (Fig. 2). Intranasal administration induces antigen-specific mucosal immune responses through the mucosal imprinting and lymphocyte homing pathway, effectively stimulating immunity in the respiratory tract [113]. Therefore, intranasal mucosal vaccines are considered a reasonable and effective strategy for preventing transmissible diseases and respiratory infections, including COVID-19 caused by SARS-CoV-2 [4, 114]. A distinctive feature of the intranasal/inhalation route is its ability to induce Th17 effector cells and tissue-resident memory (T_RM_) cells that produce IL-17 [115]. To date, only a few intranasal vaccines have been approved for clinical use, with the FluMist intranasal influenza vaccine being authorized for use in individuals aged 2 to 49 years [24]. In India, a similar intranasal influenza vaccine targeting H1N1 (Nasovac-S) has been implemented [116]. Additionally, some other promising studies have shown that intranasal BCG vaccines demonstrate good efficacy in preventing tuberculosis [117], and the BPZE1 intranasal pertussis vaccine is capable of inducing broad and specific mucosal IgA responses [118, 119].

Additionally, oral vaccines can induce robust immune responses in the salivary glands, and sublingual vaccination has been shown to provoke immune responses in both the upper and lower respiratory tracts [113]. Given the reduced efficacy of current vaccines against emerging variants of respiratory pathogens, mucosal administration routes have gained particular importance due to their advantages in terms of convenience and patient acceptance.

In addition to selecting appropriate immunization routes, the formulation and delivery strategies of vaccines are equally critical. For instance, the nasal cavity features a large mucosal surface area and is rich in immune cells, making it an ideal site for non-invasive vaccine administration via aerosol sprays[120]. Oral vaccines, however, face significant challenges due to gastric acid and digestive enzymes, which can lead to antigen degradation. To overcome this, enteric-coated capsules or microparticle technologies can be employed to protect the antigen and ensure its release and absorption in the small intestine [121].

### Gastrointestinal mucosal immunity

In intestinal pathogen infections, various forms of infection may arise. The infection may be invasive, such as in typhoid fever and poliomyelitis [122]; partially or locally invasive, as seen in Shigellosis; or strictly confined to the mucosa, as in cholera and enterotoxigenic Escherichia coli (ETEC) infections [5]. The immune response to pathogens in the gastrointestinal tract primarily occurs within the intestinal mucosa, which is composed of a single layer of epithelial cells, underlying connective tissue, and the underlying muscle layers. Most immune cells are distributed in the epithelial and lamina propria layers, forming specific immune compartments. Gut-associated lymphoid tissues (GALT) in both humans and mice include Peyer's patches and isolated lymphoid follicles [5, 123]. B cells and T cells facilitate the connection between inductive and effector sites through selective expression of integrins and chemokine receptors. For example, the expression of α4β7 integrin and the CCR9 receptor on lymphocytes in the small intestine is critical for the specific homing of these cells [124].

Importantly, the optimal route for inducing gastrointestinal mucosal immunity is oral administration [125]. However, evidence suggests that in some cases, intranasal vaccination may also trigger protective humoral and T cell responses in the intestine [126]. Several oral vaccines targeting intestinal infections, such as those for poliomyelitis, typhoid fever, cholera, and gastroenteritis, are currently available on the market [127].

Of particular note, the intestine, as one of the most important digestive systems, harbors a large number of symbiotic microorganisms, which significantly influence the intestinal mucosal immune response. In recent years, the concept of the gut microbiome-organ axis has gained increasing attention. This concept describes the continuous and complex signaling pathways between the host immune system and the gut microbiome. These interactions extend beyond the local intestinal environment and have far-reaching effects on the host's overall health and disease states through axes such as the gut-lung, gut-liver, gut-brain, and gut-kidney [128–130].

Studies have shown that the gut microbiota, through its diversity and composition, regulates the development and function of the host's mucosal immune system, establishing a complex network of interactions between innate and adaptive immunity, which plays a critical role in defending against pathogen infections [131, 132]. Additionally, probiotics, as live microorganisms, have been shown to alleviate infections and enhance the local and systemic immune responses to vaccines [133]. Research indicates that specific probiotics not only improve the immune response to viral vaccines like rotavirus (RV) vaccines [134], but also enhance overall immune function by promoting intestinal immunity and modulating various subsets of helper T cells (e.g., Th1, Th2, Th17, and Treg cells) [135]. The microbiome not only modulates local immune responses but also regulates metabolic and immune homeostasis through remote signaling pathways. A deeper understanding of the interactions between the gut microbiome and the immune system will contribute to the development of more efficient vaccines and therapeutic strategies, offering new insights for systemic immune regulation and the treatment of complex diseases.

### Other mucosal immunity

Mucosal vaccines for the reproductive tract hold significant promise in combating sexually transmitted diseases and local tumors, especially cervical cancer, which is the fourth most common cancer among women globally [5]. Additionally, the rising prevalence of drug-resistant sexually transmitted diseases is a growing concern, and preventive mucosal vaccine strategies may offer effective control measures [136]. Furthermore, using a recombinant influenza virus-HIV vector, combined intranasal and intravaginal vaccination routes have induced HIV-specific CD8^+^ tissue-resident memory (T_RM_) cells in the vaginal mucosa of mice. Vaginal immunization with a live-attenuated HSV-2 strain led to the generation of IFN-γ^+^ CD4^+^ T_RM_ cell populations, which, upon subsequent attack, could recruit memory B cells through the action of CXCL9 and CXCL10. In contrast, primary vaccination did not induce a resident plasma cell population in the female reproductive tract [137]. This suggests that following systemic priming, vaginal or potentially colorectal boosting vaccination could be an effective strategy to trigger immune responses in the reproductive tract. Emerging immunization routes such as sublingual and vaginal administration have garnered increasing attention in recent years. Particularly within the genital tract, vaccines delivered via sublingual or vaginal routes are more effective at eliciting localized immune responses, demonstrating promising potential for the prevention and control of sexually transmitted infections such as HIV and herpes simplex virus (HSV) [137, 138]. In these delivery modalities, soluble films or gel formulations are commonly employed to prolong antigen retention on the mucosal surface, thereby enhancing local immunogenicity [139].

In addition to vaginal immunity, other less explored immune pathways include ocular and rectal routes. The ocular mucosa shares some common immunological characteristics with other mucosal surfaces. For instance, conjunctiva-associated lymphoid tissue (CALT) contains CD4^+^ and CD8^+^ T cells, mast cells, dendritic cells (DCs), and Langerhans cells [53]. Evidence from research suggests that the ocular mucosa contains functional M cells capable of absorbing luminal antigens [140], which could serve as an effective and safe alternative immune route for targeting human papillomavirus (HPV) and influenza viruses [141]. For example, eye-drop vaccination with H1N1 influenza virus (A/PR/8) induced influenza-specific systemic and mucosal antibody responses, providing complete protection in mice against respiratory infection caused by A/PR/8 influenza virus [142]. Furthermore, due to the limited efficacy of oral vaccines in inducing S-IgA antibodies in the colon and distal female reproductive tract, an alternative immune route—rectal administration—can be employed to prevent pathogens such as human immunodeficiency virus (HIV) from invading through the distal digestive tract [4]. Likewise, compared to more conventional routes, rectal administration offers unique advantages, such as avoiding the degradation of vaccines in the stomach, which is a common challenge with oral formulations [53]. Furthermore, suppositories can form a drug reservoir on the rectal mucosa, enabling sustained antigen release. In recent years, solid lipid nanoparticles (SLNs) have been employed to encapsulate vaccines for rectal delivery, significantly enhancing immunogenicity [143]. Notably, dual-route vaccines, such as intranasal combined with vaginal immunization or intranasal combined with intramuscular immunization, have also shown promising results in activating both antigen-specific mucosal and systemic immune responses [138, 144]. Therefore, the selection of the immune route becomes a crucial consideration in the design of mucosal vaccines.

Studies have indicated that the immunization route not only influences the tissue-specific distribution of effector and memory cells but also plays a role in the tolerance to particular antigens. Stray and colleagues showed that intrauterine administration of UV-inactivated Chlamydia trachomatis (Ct), but not intranasally, triggered the induction of regulatory T cells, which increased the mice's susceptibility to subsequent Ct infections [145]. However, this induced tolerance was reversible when UV-inactivated Ct was combined with a novel charge-switching synthetic adjuvant particle (cSAP), which specifically targets immunogenic CD11b^+^CD103^+^ dendritic cells (DCs) in the uterine mucosa. Remarkably, mucosal delivery of the cSAP-UV-Ct formulation was the only route that led to the activation of effector T cells, which in turn facilitated the rapid recruitment of resident memory T cells (T_RM_) to the uterine mucosa, a response not observed following subcutaneous administration of the same formulation [53, 145].

## Technological platforms for the development of mucosal vaccines

The immunological characteristics of various mucosal sites provide critical guidance for vaccine design. Accordingly, selecting an appropriate vaccine platform is fundamental to achieving effective mucosal protection.

**Fig. 3:**
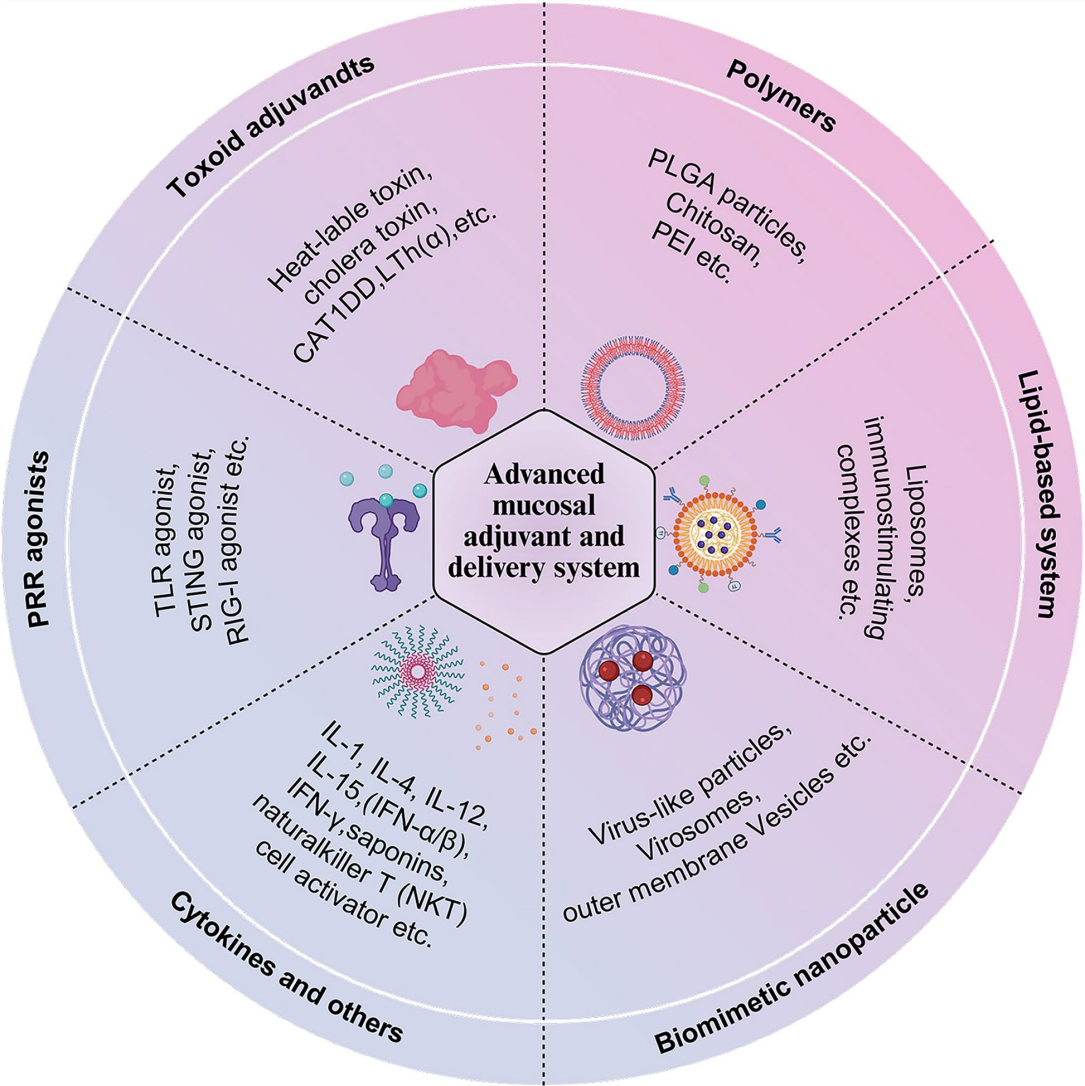
Main vaccine platforms under development for mucosal immunization. The main mucosal vaccine platforms include Attenuated/Inactivated Vaccines (top left), Subunit Protein Vaccines (bottom left), Viral Vector Vaccines (top right), and Nucleic Acid Vaccines (bottom right). Each section highlights the key features of these platforms. Each platform has distinct strengths and challenges in the context of mucosal immunity, with ongoing research focused on optimizing vaccine effectiveness and delivery

Vaccines are among the most effective tools for combating infectious diseases, making the rapid development of mucosal vaccines crucial for public health. Understanding the advantages and limitations of different mucosal vaccine platforms is of paramount importance in advancing vaccine research and development [52]. (Table 2). Although several effective mucosal vaccines are currently in use, the majority of these are live attenuated or inactivated whole-body vaccines, primarily targeting enteric pathogens. This highlights the need for further exploration into alternative mucosal vaccine strategies to address a broader range of pathogens, particularly those that affect other mucosal surfaces such as the respiratory and urogenital tracts [24]. Despite significant advancements in injectable vaccines, including adjuvanted subunit antigens, RNA, and DNA vaccines, these innovations have not yet translated into licensed mucosal vaccines on a large scale [2]. (Fig. 3).

**Table 2:** Summary of mucosal vaccine types and their characteristics

Category	route of administration	Type of induced immunity	Security	Regulatory status	Advantages	Disadvantages	Reference
Live attenuated vaccine	Intranasal (spray), Oral, Sublingual/Vaginal (preclinical)	Strong mucosal IgA, T_RM_, and systemic IgG;	Moderate (risk of reversion in immunocompromised)	Widely approved for mucosal use (FluMist, OPV)	Well-established technology; Simulating viral delivery to mucosal sites has a strong ability to penetrate epithelial barriers; May not need adjuvants	Cannot be given to immunocompromised patients; Complex manufacturing and safety requirements	[5, 146–148]
Inactivated vaccine	Intranasal (with adjuvants), Oral (needs coating), Sublingual/Rectal (experimental)	Weak IgA, requires strong adjuvants; stable but less immunogenic	High (non-replicating, stable)	Licensed (some mucosal forms, e.g., oral cholera)	Easy for large-scale production; Widely used	Differences in the effects of tropical disorders; Large-scale and expensive verification	[5, 12, 147]
Protein Subunit vaccine	Intranasal (with adjuvants), Sublingual/Vaginal/Rectal (with mucoadhesives)	High safety, low immunogenicity; often requires delivery systems	High (no live agents, good safety)	Many under trial for mucosal use	Can be lyophilized for good environmental stability.; Cannot modify host genome; Can be used regardless of age or immunocompromised status; For known immune targets	Sensitive to pH and degradation by enzymes; Inability to penetrate mucus barriers; Difficulty in isolating related antigens and complex manufacturing requirements	[5, 147, 149]
Viral vector vaccine	Intranasal, Rectal/Vaginal (preclinical), Sublingual	Strong T_RM_ and cellular responses; self-adjuvanted	Moderate (vector immunity concern)	Several in phase I–III (e.g., nasal Ad5-nCoV)	Strong ability to penetrate the mucosal barrier and strong ability to tolerate poor environments; May not need adjuvants; Adapting to rapidly mutating pathogens	Immune escape; Concerns for host genome modification/integration; Response reduced due to pre-existing immunity against the vector; Complex manufacturing and safety requirements	[5, 147, 150–153]
mRNA vaccine	Intranasal (early research), Sublingual/Rectal (experimental), Poor oral stability	Promising cellular immunity; requires lipid-based delivery systems	High (if properly encapsulated in LNPs)	Early-stage trials for mucosal use	Produces high systemic antibody titers; Cannot modify host genome; Short development and manufacturing time	Rely on advanced technology and equipment; Adjuvants are required to break mucosal immune tolerance; Difficult to penetrate the mucus barrier	[5, 147, 154, 155]

### Attenuated live vaccines

The live attenuated vaccines are made from weakened versions of infectious pathogens, produced through physical, chemical, or biological methods. These vaccines contain antigens that closely resemble those found in actual infections [156]. Approved live attenuated vaccines include oral poliovirus live attenuated vaccines (OPV), bOPV, mOPV, tOPV, live attenuated typhoid Salmonella vaccines, Rotarix (live attenuated monovalent human rotavirus vaccine), and RotaTeq (pentavalent recombinant rotavirus vaccine). Live attenuated vaccines offer several immunological advantages for mucosal vaccination. Unlike antigen-only formulations, live attenuated vaccines simulate the viral delivery to mucosal sites, which is crucial for overcoming epithelial barriers [157].

However, a major drawback of live attenuated vaccines is that since the pathogen is live, there is a possibility of reversion to the wild-type strain, or secondary mutations may transform the attenuated vaccine strain into a more infectious and virulent form, making it unsuitable for individuals with weakened immune systems [5, 146]. For example, intranasal live attenuated influenza vaccines have been linked to rare episodes of acute asthma exacerbations [156]. Issues have also been observed with parenteral live attenuated influenza vaccines, though they are now generally considered safe for asthma patients. Common side effects of oral cholera vaccines include fatigue, headaches, and gastrointestinal symptoms [158, 159]. The incidence of vaccine-associated paralytic poliomyelitis is 4.7 cases per million newborns [160]. Additionally, for intranasal live attenuated vaccines, there is a potential risk of entering the central nervous system through the olfactory bulb, leading to side effects such as encephalitis, although this has not yet been confirmed [161]. Thus, the urgent need for safer mucosal vaccines, such as inactivated vaccines, subunit vaccines, and nucleic acid vaccines, is apparent.

### Inactivated vaccines

Inactivated whole-pathogen vaccines are made by heating or chemically inactivating the pathogens (e.g., inactivated poliovirus (Salk) and hepatitis A), making them non-infectious and generally safe. It has been observed that inactivated vaccines induce a weaker short-term immune response, necessitating booster doses for full protection [162]. Inactivated vaccines are typically more stable and require simpler storage conditions [156]. They can also be lyophilized and stored at ambient temperatures for extended periods without compromising stability or efficacy, as seen with inactivated whole-cell cholera vaccines containing the B subunit of cholera toxin [163].

Researchers have developed intranasal mucosal immunization vaccines designed to induce localized immune responses in the respiratory tract via the nasal route, aiming to prevent viral entry and transmission. Significant progress has been made in injectable inactivated vaccines targeting the novel coronavirus (SARS-CoV-2) [157, 164]. However, the complexity of mucosal immunity and the presence of mucosal barriers pose challenges to the application of inactivated vaccines through mucosal routes. Studies indicate that limited antigen exposure may lead to poor immunogenicity, especially for highly conserved epitopes. These epitopes typically induce broader immune responses, and most immune reactions in nature are subdominant. For example, the hemagglutinin stalk domain of the influenza virus or the envelope proteins and fusion peptides of type 1 viral fusion proteins exhibit such characteristics [57, 165–167]. To address these challenges, adjuvants can be incorporated into inactivated vaccines to enhance efficacy by stimulating immune responses or modulating antigen presentation. However, this approach carries potential risks, including increased reactogenicity and inflammation.

A significant advantage of inactivated vaccines vaccine platforms and other non-replicating vaccine platforms is that anti-vector immunity does not become an obstacle when using the same platform for multiple vaccines [168], unlike viral vector vaccines (e.g., adenovirus or vaccinia virus vector vaccines), making it challenging to use the same viral vector for multiple disease antigens [169, 170]. For inactivated mucosal vaccines, current research is focused on improving vaccine delivery efficiency, enhancing immunogenicity, and overcoming mucosal barriers. In the future, with advancements in bioengineering and immunological research, inactivated mucosal vaccines are expected to play a larger role in preventing a range of diseases transmitted via mucosal routes.

### Subunit vaccines

One particularly effective approach to mucosal immunity involves the use of subunit vaccines combined with appropriate adjuvants and delivery systems to induce immune responses via mucosal routes, such as nasal sprays or oral administration. Similar to inactivated whole pathogen vaccines, purified or recombinant subunit vaccines do not contain live components of the pathogen but are composed solely of antigenic parts of the pathogen. This distinction makes subunit vaccines different from whole-cell vaccines [162]. Subunit vaccines are generally considered safe in terms of toxicity and reactogenicity because they contain purified or recombinant antigens rather than entire cells [149].

A notable advantage of subunit vaccines is that they can be designed to target highly conserved antigenic parts of the pathogen, enabling the development of variant-resistant vaccines and offering protection against a broad class of pathogens. This feature is especially useful for rapidly mutating pathogens, such as those infecting individuals (e.g., HIV) or those that cross populations (e.g., coronaviruses). Another advantage is that subunit vaccines target known immune targets on the pathogen, such as viral envelope proteins or pathogenic microbial toxins, thereby providing protective immunity. For example, viral envelope proteins involved in viral particle binding and host cell entry, or microbial toxins responsible for disease pathogenesis [156]. Subunit vaccines also have several highly successful examples, such as the hepatitis B virus (HBV) vaccine. Additionally, during the COVID-19 pandemic, Shifa Pharmed in Iran developed the COVIran Barakat vaccine, which received emergency use authorization in Iran [171]. Despite these successes, subunit vaccines generally suffer from poor immunogenicity and face challenges during delivery, such as the mucosal barrier and immunosuppressive microenvironments. Therefore, novel adjuvants and improved delivery systems are often required to enhance their protective potential.

First, the addition of substances with good adhesive properties, such as chitosan, engineered grains (e.g., MucoRice) [172], or starch-based microspheres (e.g., Spherex), can effectively promote the attachment of the drug to the mucosal surface, thereby prolonging its action time and improving its absorption rate. Additionally, some polymers, such as Carbopol and sodium alginate, or cationic nanogels, also possess good adhesion and biocompatibility, making them suitable for mucosal delivery [173, 174]. The use of immune stimulants is also a key strategy, particularly adjuvants that can activate the immune system and enhance the immune response to vaccines or therapeutic drugs. At the same time, the addition of innate receptor agonists can further modulate immune responses, providing more effective protection. Moreover, the application of permeation enhancers, such as bacterial toxins (e.g., cholera toxin [CT] and heat-labile enterotoxin [LT]), can increase the permeability of the mucosal barrier, thereby enhancing drug absorption [6]. Specific details will be discussed further in the subsequent section.

### Viral vector vaccines

Viral vector vaccines use genetically modified, harmless viruses as carriers to deliver genetic information (DNA or RNA) encoding the antigen into human cells, simulating the natural process of viral infection to stimulate the desired immune response [170]. As one of the promising strategies for mucosal vaccination, viral vector vaccines offer unique advantages over traditional vaccines. They not only induce strong antibody responses but also effectively stimulate T cell responses, which are crucial for the clearance of intracellular pathogens [150–152]. This characteristic is primarily attributed to the intracellular delivery capability, multifunctionality, and inherent immunogenicity of viral vectors. Furthermore, viral vectors such as adenovirus, modified Ankara vaccinia (MVA), and vesicular stomatitis virus (VSV) have been successfully used in the production of vaccines targeting pathogens such as Ebola virus [175, 176]. The basic principles behind using viruses to deliver "vaccine genes" involve several aspects. First, viral vector vaccines are considered safe, as they induce both innate and adaptive immune responses without involving fully pathogenic microorganisms. Secondly, due to the expression of various pathogen-associated molecular patterns (PAMPs) and the activation of innate immunity, viral vectors possess inherent adjuvant properties. Additionally, viral vectors can be engineered to deliver antigens to specific cells or tissues. They can also be modified to be either replication-competent or replication-deficient, enhancing their safety while reducing reactogenicity. Notably, viral vector vaccines can recapitulate the natural infection process of specific pathogens, thereby triggering classical acute inflammation and immune detection through the natural production of PAMPs, which facilitates mucosal delivery and the induction of both local mucosal and systemic immunity [177].

It is noteworthy that, due to the COVID-19 pandemic, there has been rapid advancement in both preclinical and clinical research on viral vector delivery platforms, particularly adenoviral vectors [178]. However, pre-existing anti-adenoviral immunity in humans may reduce the vaccine's efficacy. To address this, several strategies have been explored, including the use of rare adenoviruses with lower human seroprevalence (such as HuAd), chimeric adenoviruses, and adenoviruses derived from non-human species (e.g., chimpanzee-derived ChAd) [170, 179]. These optimizations have improved vaccine immunogenicity by reducing interference from host antibodies. Furthermore, adenoviral vector vaccines delivered via the respiratory mucosa have been shown to bypass interference from pre-existing antibodies in circulation [178, 180].

Currently, optimization strategies for vaccination focus on extending the interval between primary and booster vaccinations or using heterologous viral vector combinations to circumvent pre-existing immunity, thereby further enhancing vaccine efficacy. Different viral vectors exhibit significant variations in antigen expression kinetics, immune response strength, and quality. For example, modified Ankara vaccinia (MVA) vector vaccines accelerate the CD8 T-cell response with a central memory phenotype, whereas adenovirus (AdV) vaccines allow for sustained antigen delivery, inducing effector memory T-cell responses [178, 181]. Furthermore, MVA and AdV vaccines show differences in the induction of type I interferon (IFN) gene expression and subsequent immune responses, which may influence the vaccine's immunogenicity and safety [182, 183]. Based on safety and robust immunogenicity, recombinant viral vector vaccines, such as the replication-deficient chimpanzee-derived adenovirus (ChAd) developed by the University of Oxford, have become attractive candidates due to their low seroprevalence in populations and superior immunogenicity compared to human adenoviruses [184]. However, it was unexpectedly found to be less successful in experiments when used as a representative adenovirus.

Despite substantial preclinical studies supporting the development of mucosal delivery viral vector vaccines, research on their immunogenicity, protective efficacy, and potency in humans remains limited. For example, MVA and HuAd5 vector vaccines delivered via deep lung aerosol routes have demonstrated superior performance in generating mucosal immune responses. However, whether similar responses can induce strong mucosal antibody and resident B-cell memory responses to future pathogens requires further investigation [179, 180, 185]. Existing studies have found that in individuals receiving the CoronaVac (inactivated SARS-CoV-2 vaccine) as the primary vaccination, a booster dose of HuAd5 vector vaccine delivered via deep lung aerosol significantly enhanced circulating cross-neutralizing antibody levels, but the mechanisms underlying local mucosal immune responses remain incompletely understood [186]. Further exploration of the mechanisms of viral vector vaccines through respiratory mucosal delivery is crucial for addressing globally important respiratory pathogens [187, 188].

Additionally, numerous viral vectors have been explored for the delivery of mucosal antigen genes. For instance, a research team from China (including Hong Kong University, Xiamen University, and Beijing Wantai Biopharmaceutical Co.) developed an innovative nasal vaccine, dNS1-RBD, using a live attenuated influenza virus with a deleted NS1 gene as a vector to express the receptor-binding domain (RBD) of SARS-CoV-2. This vaccine received emergency use approval in China in December 2022 [189]. Mucosal vaccines using vectors such as Newcastle disease virus (NDV) [190], parainfluenza virus (PIV) [191], and attenuated respiratory syncytial virus (RSV) [192] are also in clinical trials. Other potential vaccine platforms, such as modified influenza virus vectors, Sendai virus, lentivirus [193], vesicular stomatitis virus (VSV), and recombinant rhesus macaque cytomegalovirus (RhCMV) vectors [194–196], although mostly still in the preclinical research phase, offer promising options for the development of future mucosal vaccines.

### Nucleic acid vaccines (DNA/RNA)

Although nucleic acid technologies (such as DNA/mRNA) have been widely studied since the 1990 s, it was not until the SARS-CoV-2 pandemic that mRNA vaccines were first authorized for human use. To date, no nucleic acid-based mucosal vaccines have been successfully used in humans. This is primarily due to the mucosal tolerance to live or whole-cell antigens, as well as the susceptibility of nucleic acids to degradation by enzymes, chemicals, or microbiomes. Additionally, challenges such as poor exposure to the mucosal layer, rapid nucleic acid degradation, and low cell uptake or transfection efficiency persist [197]. Currently, mRNA vaccines are predominantly administered via intramuscular injection, inducing robust systemic immunity. While they are effective in enhancing resistance to systemic infections and reducing the risk of severe disease, they may fall short in addressing mucosal infections [154].

Studies have shown that an efficient delivery system is crucial for the translation, immunogenicity, and efficacy of mucosal mRNA vaccines. To ensure that mRNA is efficiently delivered to target cells in the body, researchers have explored various delivery strategies. These strategies aim to protect the mRNA from degradation by nucleases, maximize the delivery of mRNA to target cells, and ensure its effective transportation to the cytoplasm [154]. Common delivery carriers include lipid-based, polymer-based, or hybrid delivery systems, with lipid-based carriers (such as lipid complexes or lipid nanoparticles, LNPs) being widely used due to their high efficiency [155, 198].

In the development of mucosal mRNA vaccines, it is necessary to design LNP delivery systems with mucosal adaptability. LNPs are composed of a single layer of lipid combined with surfactants, and their core components can be a combination of liquid lipids (such as LNPs), solid lipids (such as SNPs), or nanostructured lipid carriers (NLCs). By introducing modifications such as ionizable lipids, LNPs can better control the formulation of mucosal mRNA vaccines [199]. LNPs are internalized by antigen-presenting cells through receptor-mediated endocytosis. Once inside the cell, the decrease in pH within the endosome causes the protonation of ionizable lipids, promoting the fusion of the LNP membrane and releasing the mRNA into the cytoplasm, thereby driving a robust immune response. Compared to traditional liposomes, the lipid membrane of LNPs can undergo additional modifications on both the drug surface and the carrier itself, thereby improving drug delivery efficiency and immune responses [199].

Based on these characteristics, LNP (lipid nanoparticle) systems are currently considered the most effective delivery platform. Their basic components include cationic/ionizable lipids, cholesterol, polyethylene glycol (PEG) lipids, and phospholipids. The synergistic action of these components ensures the effective protection, uptake, endosomal escape, and translation of mRNA molecules, thereby driving robust cellular and humoral immune responses [155, 198, 200–202]. Furthermore, studies have indicated that the proposal of using exosome vehicles (EVs) derived from edible plants to carry mucosal mRNA vaccines appears to be feasible. This approach does not require any adjuvants, presents no safety concerns in experiments, and elicits a satisfactory mucosal immune response in both the gastrointestinal and respiratory tracts [154]. In addition, polymer-based and hybrid delivery systems are also widely discussed and applied, with several polymer biomaterials being explored, such as polyamines (polyethyleneimine, PEI) [30], polyesters (polyhydroxyalkanoates, PHA; poly(lactic-co-glycolic acid), PLGA; poly(β-amino ester), PBAE) [31]. These polymers, used in various stoichiometries, can control different vaccine properties and may be applied to the development of mucosal vaccines in the future.

The efficacy of mucosal vaccines largely depends on the use of suitable adjuvants and delivery systems. The following section highlights key technological strategies for enhancing mucosal vaccine performance.

## Advanced mucosal adjuvant and delivery system

Building on the detailed exploration of various mucosal vaccine technology platforms and their application advantages and limitations, this section further introduces novel mucosal adjuvants and advanced delivery systems that have garnered significant attention in recent years. These include toxin-based adjuvants, pattern recognition receptor (PRR) agonists, cytokine-based adjuvants, and nanoparticle-based delivery carriers. These innovative adjuvants and delivery systems substantially expand the scope and potential of mucosal vaccine development by enhancing local antigen delivery efficiency, immunogenicity, and durability of immune responses. Therefore, this comprehensive discussion provides crucial technical support and theoretical guidance to overcome the current bottlenecks in vaccine efficacy.

### Adjuvants

**Fig. 4:**
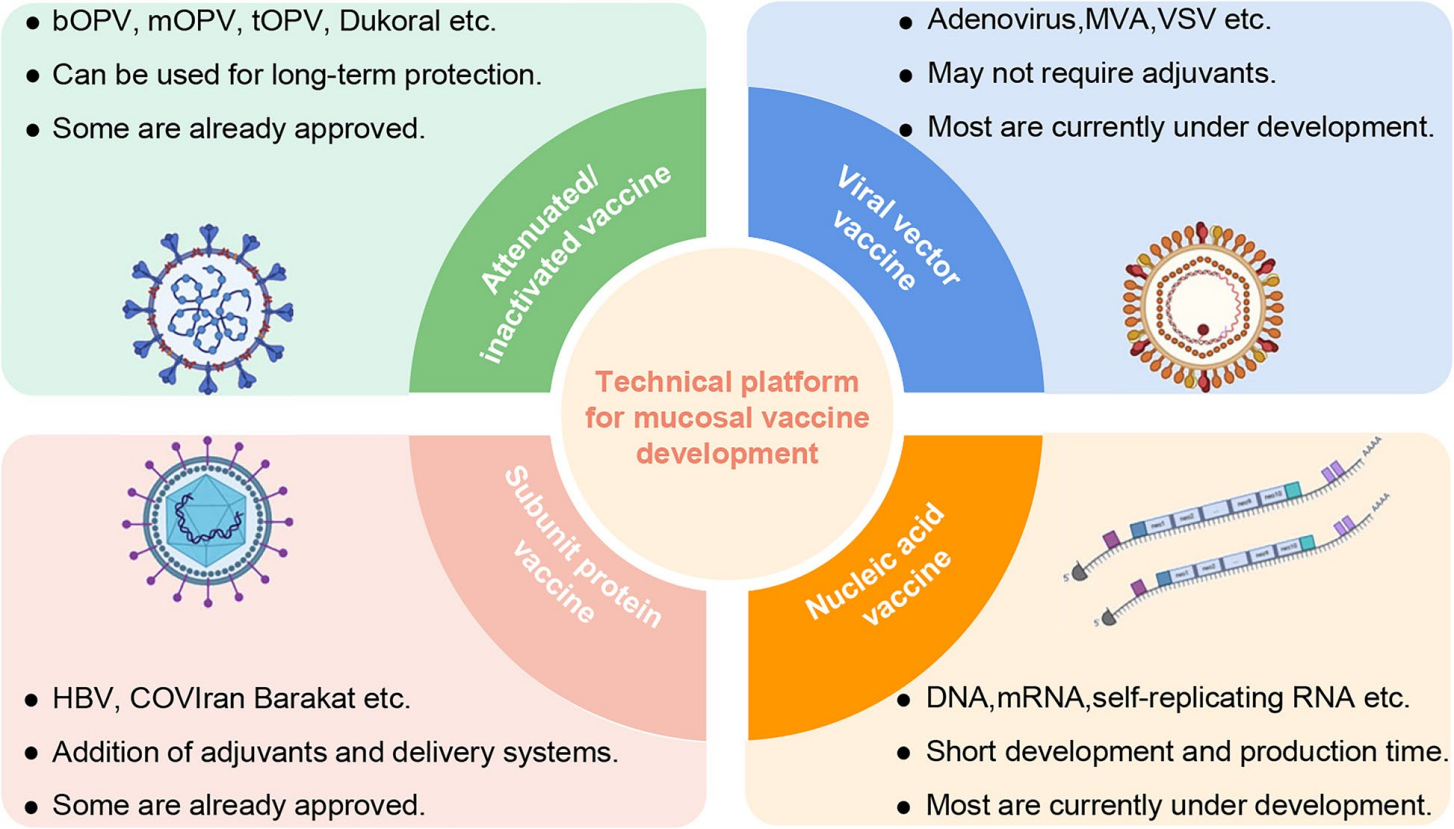
Advanced mucosal vaccine adjuvants and delivery systems. Advanced mucosal vaccine adjuvants and delivery systems include toxoid adjuvants (e.g., cholera toxin), pattern recognition receptor (PRR) agonists (e.g., TLR, STING), cytokines (e.g., IL-12, IL-15), polymers (e.g., PLGA, chitosan), lipid-based systems (e.g., liposomes), and biomimetic nanoparticles (e.g., virus-like particles). Each category enhances immune responses at mucosal surfaces, thereby improving vaccine efficacy and providing robust immune protection

One of the biggest bottlenecks in the development of mucosal vaccines is how to efficiently deliver antigens to the local mucosal site to induce mucosal immune responses. Therefore, finding safe and effective adjuvants and drug delivery systems has naturally become the focus of attention [203]. This article discusses some advanced mucosal adjuvants and delivery systems (Fig. 4). Generally, mucosal adjuvants serve two primary functions: first, they act as immune-stimulatory molecules, and second, they serve as delivery carriers. Mucosal adjuvants mainly include toxoid-like adjuvants, pattern recognition receptor (PRR) agonists, cytokine-based adjuvants, and other mucosal adjuvants [12, 24] (Table 3).

**Table 3:** Advanced adjuvant strategies involved in mucosal immune responses

Class	Molecule/Mechanism	Immune Cell Target
Toxoid-like	Cholera Toxin	Dendritic cells, CD4^+^ T cells
	*E. coli* heat-labile toxin	Dendritic cells, Macrophages
	Double-mutant Labile Toxin	Dendritic cells, Macrophages, M cells
	Multi-mutation CT	CD4^+^ T cells, CD8^+^ T cells, Dendritic cells, NK cells, Macrophages, B cells
	Cholera Toxin A1-dimer D-domain (S. aureus)	Dendritic cells, Macrophages, CD4^+^ T cells
	LThαK	CD4^+^ T cells, CD8^+^ T cells, Dendritic cells, NK cells, Macrophages
TLR ligands	MPL–TLR4	Dendritic cells, Macrophages
	CpG–TLR9	B cells, Plasma cells
	Flagellin–TLR5S	Dendritic cells, Macrophages
	TLR7/8–Imidazoquinolinone derivatives	Dendritic cell, Macrophages, NK Cells, B-cells, CD4^+^ T cells, CD8^+^ T cells
Cytokines	IL-1, IL-4, IL-12, IL-15, IL-17, IL-18, IFN-α/β, IFN-γ, IFN-λ, GM-CSF, RANTES	CD8^+^ T cells, B cells (IgA), Monocytes, Natural Killer cells, CD4^+^ and CD8^+^ T cells
Chitosan	Mucoadhesive, improves antigen uptake	Dendritic cell, Macrophages, Natural Killer cells
Saponin complexes, ISCOM, QS-21, Matrix-M	Induction of humoral immunity	Th1, Th2 and CD8^+^ T-cells
α-Galactosyl ceramide	CD1 binding	Dendritic cell, CD8^+^ T cells
VLP, Virosomes, Liposomes (IRIV)	PAMP signals; Improved APC antigen uptake to promote	Dendritic cells, B-cells, T cells

Toxoid-like adjuvants: These include heat-labile enterotoxin (LT) from *Escherichia coli* and cholera toxin (CT). Due to their strong adjuvant properties and ability to induce secretory IgA (S-IgA), they are considered the "gold standard" for oral vaccination [204]. Although bacterial enterotoxins and their derivatives hold high potential as effective mucosal adjuvants, their toxicity limits their widespread clinical use [58]. For example, LT can accumulate in the olfactory bulb and other neural tissues, leading to the occurrence of Bell's palsy in vaccinated individuals [24]. To reduce toxicity, researchers have gradually developed modified versions of these toxins, such as double-mutant LT (dmLT) and multiple-mutant CT (mmCT), which retain adjuvant properties while reducing toxicity [162, 205, 206]. Furthermore, the results of a Phase II clinical trial for a trivalent influenza vaccine containing hemagglutinin and LThαK (a detoxified derivative of *E. coli* heat-labile enterotoxin) have been reported (NCT03784885). This intranasal LThαK-adjuvanted influenza vaccine was shown to be both effective and safe in clinical trials [207, 208]. Additionally, some toxin subunits or other modified or mutated toxins are used in licensed vaccines, such as the recombinant cholera toxin B subunit used in Dukoral® [209].

Apart from that, PRRs include receptors targeting Toll-like receptors (TLRs), RIG-I-like receptors (RLRs), STING, C-type lectin receptors (CLRs), and NOD-like receptors (NLRs). These adjuvants activate immune cells through pathogen-associated molecular patterns (PAMPs) to produce enhanced immune responses mediated by TLRs [12, 210]. Among these, TLR agonists have attracted significant attention as a central subgroup of mucosal adjuvants [211–213]. For example, a low-toxicity TLR4 agonist derived from LPS, monophosphorylated lipid A (MPL), is included in licensed adjuvants AS01, AS02, and AS04, and is currently undergoing preclinical studies and clinical evaluations as a mucosal adjuvant for norovirus vaccines [214, 215]. Other TLR ligands tested in mucosal vaccines include: TLR3-specific double-stranded RNA analog poly I: C; TLR5-specific flagellin; TLR7/8 agonists such as imidazoquinolinone derivatives; and TLR9 agonists such as CpG oligonucleotides, all of which have shown potential as mucosal adjuvants [211, 216–219].

Cytokine-based adjuvants, including IL-1, IL-4, IL-12, IL-15, IL-18, IL-17, type I interferons (IFN-α/β), IFN-γ, and IFN-λ, have been explored as potential mucosal adjuvants. For example, IL-12 and IL-15 help induce cytotoxic T lymphocyte (CTL) responses and antigen-specific IgA antibody production at mucosal sites, while IL-1 induces both IgA and IgG production [220, 221]. Preclinical studies have shown that combining influenza subunit vaccines with type I interferons as mucosal adjuvants significantly enhances antigen uptake by nasal mucosal phagocytes following intranasal immunization.

Other promising mucosal adjuvants include saponins (e.g., QS-21) [222], natural killer T (NKT) [223] cell activators (e.g., α-GalCer [224] and analogs KBC-007, KBC-009 [225]), protease-activated receptor 2 (PAR-2) agonists [226], and cationic nanoparticles (such as chitosan, N-[1-(2,3-diethoxy)propyl]-N, N, N-methyl chloride ammonium (DOTAP), polyethyleneimine (PEI), and recently designed cationic cross-linked carbon dot (CCD) adjuvants) [227–230]. During the development of novel vaccine adjuvants, early-stage adjuvants were unable to enter clinical development due to high reactivity and poor tolerance. Therefore, to accelerate vaccine development, selecting materials that have undergone clinical evaluation as mucosal adjuvants is crucial. For example, alum adjuvant vaccines, MF59 emulsion-adjuvanted influenza vaccines, emulsion-based vaccine adjuvants (such as AS03) combined with influenza vaccines, and AS04, a composite adjuvant containing MPLA adsorbed onto alum, have all been approved for use [3, 231–233]. In conclusion, the development of novel mucosal vaccine adjuvants with high safety, good tolerance, and strong immune responses remains a significant challenge.

### Mucosal delivery system

Various mucosal vaccine delivery systems must overcome multiple barriers, which can impact the effective induction of local immune responses. Therefore, the development of efficient delivery systems is critical. Currently, various excellent delivery systems have been designed to enhance mucosal immune efficacy [179]. An ideal mucosal vaccine delivery system must be safe, non-toxic, and capable of efficiently facilitating the uptake and presentation of antigens. Targeting M cells and DCs is a potential strategy, along with overcoming the mucus barrier by designing carriers that disrupt the mucus layer to promote antigen uptake. The carrier must also protect the antigen from enzymatic or chemical degradation to ensure safe delivery. Ultimately, vaccines should be designed according to the specific characteristics of different mucosal sites to induce specific and durable immune responses. Mucosal delivery systems typically utilize nanoparticle technology, biomaterial-based delivery, and viral vector delivery. This discussion focuses primarily on nanoparticle delivery systems: polymeric nanoparticles, lipid-based nanoparticles, and biomimetic nanoparticle systems [234].

Polymeric nanoparticles (PNPs): Polymeric nanoparticles (PNPs) are nanocarriers composed of natural or synthetic polymers. These particles can load various antigens via mechanisms such as covalent coupling, adsorption, and encapsulation, providing the advantage of easy generation and highly flexible structural modifications [235, 236]. The molecular weight and chemical structure of the polymer material can be adjusted to precisely control its physicochemical properties, achieving the desired characteristics. Synthetic polymer nanoparticles include particulate delivery agents that encapsulate antigens and can target specific tissues by imparting diverse physicochemical properties and binding capabilities. Among these, PLGA (poly (lactic-co-glycolic acid)) is a widely studied polymer, with its encapsulated TLR agonists inducing strong antigen-specific mucosal immune responses against various pathogens [237]. However, PLGA particles exhibit some limitations, such as low bioadhesion [238–240]. To address this issue, chitosan has been introduced to enhance the mucus adhesion of PLGA particles, thereby improving the mucosal immunogenicity of the vaccine [241]. Another type of synthetic polymer nanoparticle is dendritic polymers, which consist of a central core surrounded by symmetrically branched nanostructures with a monodisperse structure. Due to their dendritic structure, these polymers form internal cavities and can be customized by introducing functional surface moieties to alter their physicochemical and biological properties [242]. Natural polymer nanoparticles that display bio-adhesive properties include chitosan, maltodextrin, alginates, hyaluronic acid, carboxymethyl cellulose, hydroxyethyl cellulose, and pectin [243]. Specifically, chitosan is a natural polysaccharide with adhesive, permeable, and biodegradable properties. By loosening tight junctions between epithelial cells, chitosan enhances the uptake of antigens at the mucosal surface, making it an ideal polymer for mucosal vaccine delivery.

Lipid-Based Nanoparticle Systems: Lipid nanoparticles (LNPs) are nanoscale systems (< 1 μm) composed of two or more (usually four) different proportions of lipids. They can form various structures and are currently the most successful non-viral nanocarriers, suitable for clinical translation. LNPs offer several advantages, such as high encapsulation efficiency, low toxicity, enhanced cellular uptake, and high stability, making them a representative and relatively mature carrier system [244]. Liposomes, typically spherical vesicles composed of biodegradable, non-toxic, and non-immunogenic lipid bilayers, possess a variety of physicochemical properties, including different sizes, lipid compositions, and charges, which enable rational vaccine design [245]. Moreover, liposomes can protect antigens from degradation in the harsh mucosal environment by encapsulating them in a hydrophilic core or complexing them with acyl chains or charged surfaces [245]. Importantly, by coating or modifying the surface of liposomes with specific ligands and adjuvants, liposomes can selectively deliver antigens to specific organs, thereby inducing stronger immune responses [246, 247]. This feature enables more effective and selective immune responses against encapsulated antigens. Liposomes are commonly used in mucosal delivery systems, especially for intranasal immunization, and have been shown to be effective against pathogens such as SARS-CoV-2, influenza, Yersinia pestis, and Streptococcus pneumoniae [248–250].

Biomimetic Nanoparticle (BNP) Systems: In addition, the integration of nanotechnology and biomimetic strategies has led to the development of various nanoparticle systems. BNP platforms offer multifunctionality and hold great potential in the delivery of mucosal vaccines [234]. Among these, virus-like particles (VLPs) are considered highly promising mucosal delivery systems due to their delivery efficacy (mimicking live viruses while lacking viral genetic material) and proven safety [251]. VLPs, formed by the spontaneous self-assembly of viral coat proteins, envelope proteins, or core proteins, are able to effectively penetrate mucosal barriers and trigger a strong immune response [252]. Because of their polymer-like properties, VLPs are easily absorbed by antigen-presenting cells (APCs), particularly dendritic cells (DCs), thereby enhancing interactions with nasal-associated lymphoid tissue (NALT) cells [253]. This property makes VLP-based nasal vaccines highly promising in inducing both humoral and cellular immunity. For example, a mucosal DNA vaccine developed using phage technology stimulates the generation of VLPs resembling SARS-CoV-2 structures, leading to a robust immune response [254]. Additionally, VLP vaccines targeting influenza subtypes offer new strategies for generating broad immune responses [255]. These attributes make VLPs a powerful tool for mucosal antigen delivery, providing new opportunities for vaccine development.

Additionally, virosomes, which are recombinant spherical viral-like vesicles composed of phospholipids from viral envelope components, are gaining attention as an effective mucosal antigen delivery carrier [256]. Unlike liposomes, virosomes possess specific viral glycoproteins (hemagglutinin and neuraminidase), granting them unique immune-stimulating properties and membrane fusion capabilities. With their proven delivery efficacy and safety, virosomes are considered capable of delivering vaccine antigens directly to host cells [234]. These characteristics have made their research focus on mucosal delivery systems. Outer membrane vesicles (OMVs), which are rich in outer membrane proteins, inner membrane, and cytoplasmic proteins, also exhibit significant immune-stimulating properties and self-adjuvanticity, effectively inducing mucosal immune responses [257–260]. For instance, OMVs from Escherichia coli successfully delivered malaria antigens, and OMVs from Neisseria meningitidis delivered the SARS-CoV-2 spike protein. Following intranasal administration, these OMVs triggered robust IgA immune responses, demonstrating their effectiveness in mucosal immunity. Additionally, engineered OMVs from Salmonella typhimurium, Vibrio cholerae, and Enterotoxigenic Escherichia coli have been used to express SARS-CoV-2 spike protein and have shown significant neutralizing effects in mouse intranasal immunization [261, 262]. All of these findings further support the potential and advantages of OMVs as mucosal vaccine carriers.

## Challenges and solutions in mucosal vaccine development

Although preceding sections systematically summarize the significant progress and prospects achieved in fundamental theory, tissue specificity, and technological platforms of mucosal vaccine technology, numerous practical challenges and bottlenecks persist in actual development and application, such as safety considerations, uncertainties regarding vaccine efficacy, antigen delivery difficulties, and obstacles in clinical translation. This section focuses on these core issues, deeply analyzing existing technical and mechanistic barriers, and proposing targeted solutions, including optimizing vaccine formulations, improving delivery system design, exploring innovative immunological adjuvants, and expanding novel immunization routes. By introducing these forward-looking strategies, this section not only offers insights to overcome existing development bottlenecks but also clearly outlines pathways for the successful transition of mucosal vaccines from basic research to clinical practice.

### Development of mucosal vaccine platform technologies with high safety

Many mucosal vaccines have been developed for humans and animals, proving effective in blocking pathogen infection and transmission. Despite this, current mucosal vaccines still have limitations. As discussed earlier, we have reviewed the safety issues related to live attenuated vaccines. Compared to live attenuated vaccines, inactivated vaccines and subunit vaccines generally offer higher safety, but they require the addition of adjuvants. Some of the adjuvants currently in use face challenges related to both efficiency and safety. For instance, as little as 5 µg of purified cholera toxin (CT) can induce severe diarrhea in human volunteers, while only 2.5 µg of LT is sufficient to cause fluid secretion [53, 263]. However, several mutants, created by altering active and protease sites, have been shown to reduce toxicity while maintaining adjuvant activity [264].

Currently, the vast majority of approved mucosal vaccines are oral vaccines. For oral vaccines, the harsh gastrointestinal environment and the presence of oral tolerance mechanisms remain major challenges [265]. Additionally, attenuated live vaccines may enter the brain through the olfactory nerves, so it is necessary to evaluate the neurotropism of attenuated live viruses in order to develop intranasal vaccines [266, 267]. mRNA vaccines, which gained widespread approval during the COVID-19 pandemic, represent a promising platform for mucosal vaccine development. For mRNA-based mucosal vaccines, combining optimized delivery systems with enhanced adjuvants will be key to unlocking their full potential. Despite the significant promise and progress demonstrated by novel mucosal vaccine platforms, such as viral vectors, mRNA, and nanoparticles in preclinical studies, safety remains a critical challenge.

### Preclinical animal selection

In preclinical studies, animal models, especially mice, are commonly used in mucosal vaccine research. Although many principles of mucosal immunology are effective in mice, the same phenomena often cannot be replicated in humans [268]. For instance, the mouse vaginal mucosa is keratinized, while the human vaginal mucosa is non-keratinized, affecting permeability and immune response [269]. Additionally, there are significant differences between humans and mice in terms of immune system components (such as IgA and key innate immune receptors like TLRs) [270, 271], as well as differences in the size, number, distribution, and composition of mucosal lymphoid structures (such as Peyer's patches) across species, which can fundamentally influence the generation of immune responses [272]. These factors complicate the translation of results from animal models to human models. Moreover, unlike the highly controlled experimental environments in animal models, human variability—such as microbiota, nutritional status, and prior immune history—has been shown to affect the efficacy of mucosal vaccines [4]. Therefore, understanding the vast differences in anatomy, physiology, and immunology between species is crucial for future mucosal vaccine developmen. Thus, while animal models provide valuable insights, translating these findings to humans requires careful consideration of species differences and variability in clinical settings.

### Clinical evaluation of mucosal vaccines

Currently, there are no standardized methods to evaluate mucosal vaccine efficacy in clinical trials. This is likely due to the complexity of mucosal immunity and the unique physiological structures involved, which hinder the assessment of mucosal immune responses. For example, the uneven distribution and low levels of mucosal antibodies pose significant challenges for sampling and detection. Collecting bronchoalveolar lavage fluid to assess mucosal cell immunity is technically demanding, has low tolerance, and is not suitable for large-scale clinical studies [53]. While methods to detect mucosal antibodies, particularly in respiratory mucosal vaccines, have been established using non-invasive sampling techniques such as saliva, nasal washes, and swabs to measure IgA responses, the detection levels of specific mucosal antibodies are relatively low. This may be due to insufficient sampling capacity and low sensitivity of detection methods [273]. Evaluating mucosal cellular responses presents an even greater challenge, with low patient acceptability. Although nasal scraping and flocked swabs have been proven reliable for studying nasal cell immune responses during infections [274], these methods still require further evaluation for their applicability in clinical research. The lack of standardized methods for evaluating mucosal vaccine efficacy presents a major challenge to accurately assessing the effectiveness of mucosal vaccine development. Therefore, it is critical to establish standardized testing methods and sampling protocols for human mucosal vaccine evaluation as soon as possible.

### Future applications in the field of mucosal immunity

Mucosal vaccines have not only been widely used in the prevention and treatment of respiratory and gastrointestinal pathogens but also show significant potential in the treatment of mucosal malignancies, providing a new theoretical basis for their targeted applications. While some tumor vaccines that are already on the market have shown good results in mouse models and can induce T-cell clustering responses in humans, their therapeutic efficacy remains limited. Research indicates that this may be due to these vaccines' inability to effectively induce cytotoxic T-cell responses at mucosal sites. Given the potential of mucosal immune responses in tumor immunotherapy, enhancing mucosal immunity may improve the efficacy of anti-tumor vaccines in the future, particularly through mechanisms such as the induction of resident memory T cells (T_RM_ cells), leading to more effective local immune defenses [275, 276]. Moreover, targeting tumor neoantigens is an attractive strategy. However, due to the tumor's and patient's specific mutational burden and composition, this approach is unlikely to become a universal "one-size-fits-all" method [277, 278]. Therapeutic mucosal cancer vaccines can address these challenges by adopting personalized vaccine designs based on individualized medicinal approaches or by utilizing local antigen release for broader applications [279, 280]. While mucosal vaccines show great promise in infectious disease prevention, their role in cancer immunotherapy is also gaining traction, offering new possibilities for personalized treatments.

### Research on host receptor mucosal immune mechanisms

In the future, mucosal vaccines will become the mysterious key to breaking through the boundaries of immunology, ushering in an unprecedented immunological revolution. The future of mucosal vaccines will no longer be a single protective tool; they will not only effectively fend off the invasion of viruses, bacteria, and malignant tumors but will also leave permanent marks across the body’s immune networks, ensuring that any potential threats will find it difficult to break through these tightly guarded defenses. In summary, with the rapid advancements in immunology, molecular biology, and nanotechnology, future mucosal vaccines will not only make breakthroughs in the prevention and treatment of traditional pathogens but will also demonstrate enormous potential in tumor immunotherapy, personalized treatments, and the application of new vaccine platforms.

The mechanisms that generate effective vaccine-induced immune responses in mucosal tissues are complex and may vary depending on the mucosal surface. A deeper understanding of the innate and adaptive immune mechanisms at the molecular and cellular levels, especially the regulation of adaptive mucosal protection, is crucial to driving the development of mucosal vaccines. First, we must focus on exploring the mucosal barrier and antigen transport mechanisms. Although M cells play a key role in antigen translocation, studying other potential transport mechanisms is essential for ensuring the effective delivery of mucosal vaccines [281]. Furthermore, compared to circulating IgA, mucosal IgA antibodies exhibit complexity in various forms [282]. More research is needed to explore how these diverse immunoglobulin isotypes can be utilized for potential preventive or therapeutic applications. Regarding mucosal cellular immune components, T cells have long been recognized for their importance in mucosal immunity, but there is still a lack of strategies to initiate appropriate helper T cell responses. One well-studied aspect of different T cell types is their targeting of pathogens with varying characteristics [283]. Notably, the activation of tissue-resident immune cells is critical for initiating local immunity and promoting immune signals to migrate throughout the body. However, the differentiation, maintenance, and plasticity of tissue-resident T cells remain unclear, partly due to the difficulty of accessing T cells in tissues. Blood sampling can only capture circulating T cells, not tissue-resident T cells [284]. Therefore, understanding how host factors interact with the immune system is essential for the future design and development of mucosal vaccines.

## Conclusion and perspective

Despite remarkable successes and groundbreaking advancements in mucosal vaccine research, the field remains at a critical threshold, poised to address some of the most pressing global health challenges yet confronted by formidable scientific and translational obstacles. Mucosal surfaces, as primary pathogen entry points, underscore the importance of elucidating and harnessing mucosal immune responses to design more effective vaccines. While substantial progress has been achieved—particularly in preventing infectious diseases—mucosal vaccines still face significant limitations in consistently inducing durable local and systemic immunity. Central to overcoming these barriers is the optimization of mucosal adjuvants and delivery systems, which currently lack sufficient efficacy in eliciting robust and persistent mucosal immune responses. Enhancing mucoadhesive properties, prolonging antigen retention at mucosal sites, and improving antigen uptake remain essential priorities for future research and development.

In addition to the well-studied oral and intranasal routes, underutilized mucosal delivery pathways—such as ocular, rectal, and vaginal immunization—present promising but largely unexplored opportunities. These alternative routes offer unique immunological microenvironments and potentially less invasive administration options; however, their clinical translation is hindered by the absence of standardized formulations and robust validation in human trials. Expanding research efforts into these non-traditional delivery routes could diversify mucosal vaccine platforms, enhance immunization coverage, and better address the needs of specific populations, especially those underserved by conventional vaccination strategies.

A critical bottleneck in the clinical development of mucosal vaccines is the lack of unified regulatory standards and standardized preclinical models to evaluate mucosal immune responses in humans. Regulatory challenges, including gaps in manufacturing quality control and immunogenicity assessment criteria, have delayed the transition from bench to bedside. Moreover, current animal models often fail to accurately predict human mucosal immunogenicity and safety, particularly regarding long-term protection and local tolerance. Developing advanced humanized models and standardized immune monitoring platforms is imperative to improve the predictive power of preclinical studies, accelerate clinical trials, and ensure vaccine safety and efficacy across diverse demographic groups.

Finally, the integration of mucosal immunology with emerging fields such as microbiome research and nanotechnology holds transformative potential. The complex interplay between the mucosal immune system and host microbiota critically shapes vaccine responsiveness and safety profiles, offering opportunities for personalized immunization strategies that account for individual genetic and microbial variability. Concurrently, innovations in nanomaterials and adjuvants promise to enhance antigen delivery and targeting precision. As these multidisciplinary advances converge, mucosal vaccines are expected to overcome the so-called “tropical barrier” by delivering more effective, accessible, and affordable immunization solutions worldwide. Ultimately, mucosal vaccine technology is poised to play a pivotal role in pandemic preparedness, routine infectious disease control—including influenza and respiratory syncytial virus—and cancer immunotherapy, thereby reshaping the global vaccine landscape.

## Data Availability

The datasets used and analyzed during the current study are available from the corresponding author on reasonable request.

## References

[1] Correa VA, Portilho AI, De Gaspari E. Vaccines, adjuvants and key factors for mucosal immune response. Immunology. 2022;167(2):124–38. 10.1111/imm.13526. 35751397 10.1111/imm.13526

[2] Knisely JM, Buyon LE, Mandt R, Farkas R, Balasingam S, Bok K, et al. Mucosal vaccines for SARS-CoV-2: scientific gaps and opportunities-workshop report. NPJ Vaccines. 2023;8(1):53. 10.1038/s41541-023-00654-6. 37045860 10.1038/s41541-023-00654-6 PMC10091310

[3] Li M, Wang Y, Sun Y, Cui H, Zhu SJ, Qiu HJ. Mucosal vaccines: strategies and challenges. Immunol Lett. 2020;217:116–25. 10.1016/j.imlet.2019.10.013. 31669546 10.1016/j.imlet.2019.10.013

[4] Holmgren J, Czerkinsky C. Mucosal immunity and vaccines. Nat Med. 2005;11(4 Suppl):S45-53. 10.1038/nm1213. 15812489 10.1038/nm1213

[5] Lavelle EC, Ward RW. Mucosal vaccines - fortifying the frontiers. Nat Rev Immunol. 2022;22(4):236–50. 10.1038/s41577-021-00583-2. 34312520 10.1038/s41577-021-00583-2 PMC8312369

[6] Zhou M, Xiao H, Yang X, Cheng T, Yuan L, Xia N. Novel vaccine strategies to induce respiratory mucosal immunity: advances and implications. MedComm (2020). 2025;6(2):e70056. 10.1002/mco2.70056. 10.1002/mco2.70056 PMC11739453 39830020

[7] Moradi-Kalbolandi S, Majidzadeh AK, Abdolvahab MH, Jalili N, Farahmand L. The Role of Mucosal Immunity and Recombinant Probiotics in SARS-CoV2 Vaccine Development. Probiotics Antimicrob Proteins. 2021;13(5):1239–53. 10.1007/s12602-021-09773-9. 33770348 10.1007/s12602-021-09773-9 PMC7996120

[8] Wang Z, Lorenzi JCC, Muecksch F, Finkin S, Viant C, Gaebler C, et al. Enhanced SARS-CoV-2 neutralization by dimeric IgA. Sci Transl Med. 2021. 10.1126/scitranslmed.abf1555. 33288661 10.1126/scitranslmed.abf1555 PMC7857415

[9] Moor K, Diard M, Sellin ME, Felmy B, Wotzka SY, Toska A, et al. High-avidity IgA protects the intestine by enchaining growing bacteria. Nature. 2017;544(7651):498–502. 10.1038/nature22058. 28405025 10.1038/nature22058

[10] Szabo PA, Miron M, Farber DL. Location, location, location: Tissue resident memory T cells in mice and humans. Sci Immunol. 2019. 10.1126/sciimmunol.aas9673. 30952804 10.1126/sciimmunol.aas9673 PMC6778482

[11] Noh HE, Rha MS. Mucosal immunity against SARS-CoV-2 in the respiratory tract. Pathogens. 2024;13(2): 113. 10.3390/pathogens13020113. 38392851 10.3390/pathogens13020113 PMC10892713

[12] Song Y, Mehl F, Zeichner SL. Vaccine strategies to elicit mucosal immunity. Vaccines (Basel). 2024. 10.3390/vaccines12020191. 38400174 10.3390/vaccines12020191 PMC10892965

[13] Miteva D, Peshevska-Sekulovska M, Snegarova V, Batselova H, Alexandrova R, Velikova T. Mucosal COVID-19 vaccines: risks, benefits and control of the pandemic. World J Virol. 2022;11(5):221–36. 10.5501/wjv.v11.i5.221. 36188733 10.5501/wjv.v11.i5.221 PMC9523321

[14] Li X, Geng M, Peng Y, Meng L, Lu S. Molecular immune pathogenesis and diagnosis of COVID-19. J Pharm Anal. 2020;10(2):102–8. 10.1016/j.jpha.2020.03.001. 32282863 10.1016/j.jpha.2020.03.001 PMC7104082

[15] Russell MW, Moldoveanu Z, Ogra PL, Mestecky J. Mucosal immunity in COVID-19: a neglected but critical aspect of SARS-CoV-2 infection. Front Immunol. 2020;11: 611337. 10.3389/fimmu.2020.611337. 33329607 10.3389/fimmu.2020.611337 PMC7733922

[16] Freeman D, Lambe S, Yu LM, Freeman J, Chadwick A, Vaccari C, et al. Injection fears and COVID-19 vaccine hesitancy. Psychol Med. 2023;53(4):1185–95. 10.1017/S0033291721002609. 34112276 10.1017/S0033291721002609 PMC8220023

[17] Collaborators GL. Age-sex differences in the global burden of lower respiratory infections and risk factors, 1990–2019: results from the Global Burden of Disease Study 2019. Lancet Infect Dis. 2022;22(11):1626–47. 10.1016/S1473-3099(22)00510-2. 35964613 10.1016/S1473-3099(22)00510-2 PMC9605880

[18] Mettelman RC, Allen EK, Thomas PG. Mucosal immune responses to infection and vaccination in the respiratory tract. Immunity. 2022;55(5):749–80. 10.1016/j.immuni.2022.04.013. 35545027 10.1016/j.immuni.2022.04.013 PMC9087965

[19] Feemster K, Weaver J, Buchwald U, Banniettis N, Cox KS, Mcintosh ED, Spoulou V. Pneumococcal Vaccine Breakthrough and Failure in Infants and Children: A Narrative Review. Vaccines-Basel. 2023;11(12). ARTN 1750. 10.3390/vaccines11121750. 10.3390/vaccines11121750 PMC10747311 38140155

[20] Schrager LK, Vekemens J, Drager N, Lewinsohn DM, Olesen OF. The status of tuberculosis vaccine development. Lancet Infect Dis. 2020;20(3):e28–37. 10.1016/S1473-3099(19)30625-5. 32014117 10.1016/S1473-3099(19)30625-5

[21] Kumari R, Sharma SD, Kumar A, Ende Z, Mishina M, Wang Y, et al. Antiviral approaches against influenza virus. Clin Microbiol Rev. 2023;36(1): e0004022. 10.1128/cmr.00040-22. 36645300 10.1128/cmr.00040-22 PMC10035319

[22] Qiu J, Zhang S, Feng Y, Su X, Cai J, Chen S, et al. Efficacy and safety of hepatitis B vaccine: an umbrella review of meta-analyses. Expert Rev Vaccines. 2024;23(1):69–81. 10.1080/14760584.2023.2289566. 38055218 10.1080/14760584.2023.2289566

[23] Kamolratanakul S, Pitisuttithum P. Human papillomavirus vaccine efficacy and effectiveness against cancer. Vaccines. 2021. 10.3390/vaccines9121413. 34960159 10.3390/vaccines9121413 PMC8706722

[24] Dotiwala F, Upadhyay AK. Next generation mucosal vaccine strategy for respiratory pathogens. Vaccines (Basel). 2023. 10.3390/vaccines11101585. 37896988 10.3390/vaccines11101585 PMC10611113

[25] Neutra MR, Kozlowski PA. Mucosal vaccines: the promise and the challenge. Nat Rev Immunol. 2006;6(2):148–58. 10.1038/nri1777. 16491139 10.1038/nri1777

[26] Bischoff SC. Physiological and pathophysiological functions of intestinal mast cells. Semin Immunopathol. 2009;31(2):185–205. 10.1007/s00281-009-0165-4. 19533134 10.1007/s00281-009-0165-4

[27] Mitchison HM, Valente EM. Motile and non-motile cilia in human pathology: from function to phenotypes. J Pathol. 2017;241(2):294–309. 10.1002/path.4843. 27859258 10.1002/path.4843

[28] Raemdonck K, Martens TF, Braeckmans K, Demeester J, De Smedt SC. Polysaccharide-based nucleic acid nanoformulations. Adv Drug Deliver Rev. 2013;65(9):1123–47. 10.1016/j.addr.2013.05.002. 10.1016/j.addr.2013.05.002 23680381

[29] Thompson M, Scholz C. Highly branched polymers based on poly(amino acid)s for biomedical application. Nanomaterials. 2021. 10.3390/nano11051119. 33925961 10.3390/nano11051119 PMC8145254

[30] Wibowo D, Jorritsma SHT, Gonzaga ZJ, Evert B, Chen SX, Rehm BHA. Polymeric nanoparticle vaccines to combat emerging and pandemic threats. Biomaterials. 2021;268. ARTN 120597. 10.1016/j.biomaterials.2020.120597. 10.1016/j.biomaterials.2020.120597 PMC7834201 33360074

[31] Niza E, Ocaña A, Castro-Osma JA, Bravo I, Alonso-Moreno C. Polyester polymeric nanoparticles as platforms in the development of novel nanomedicines for cancer treatment. Cancers (Basel). 2021;13(14): ARTN 3387. 10.3390/cancers13143387. 10.3390/cancers13143387 PMC8304499 34298604

[32] Baindara P, Ganguli S, Chakraborty R, Mandal SM. Preventing respiratory viral diseases with antimicrobial peptide master regulators in the lung airway habitat. Clinics Pract. 2023;13(1):125–47. 10.3390/clinpract13010012. 10.3390/clinpract13010012 PMC9844411 36648852

[33] Hill DB, Button B, Rubinstein M, Boucher RC. Physiology and pathophysiology of human airway mucus. Physiol Rev. 2022;102(4):1757–836. 10.1152/physrev.00004.2021. 35001665 10.1152/physrev.00004.2021 PMC9665957

[34] France MM, Turner JR. The mucosal barrier at a glance. J Cell Sci. 2017;130(2):307–14. 10.1242/jcs.193482. 28062847 10.1242/jcs.193482 PMC5278669

[35] Elphick DA, Mahida YR. Paneth cells: Their role in innate immunity and inflammatory disease. Gut. 2005;54(12):1802–9. 10.1136/gut.2005.068601. 16284290 10.1136/gut.2005.068601 PMC1774800

[36] Kurashima Y, Goto Y, Kiyono H. Mucosal innate immune cells regulate both gut homeostasis and intestinal inflammation. Eur J Immunol. 2013;43(12):3108–15. 10.1002/eji.201343782. 24414823 10.1002/eji.201343782

[37] Galli SJ, Tsai M, Piliponsky AM. The development of allergic inflammation. Nature. 2008;454(7203):445–54. 10.1038/nature07204. 18650915 10.1038/nature07204 PMC3573758

[38] Zhang XX, Zhang JL, Chen S, He Q, Bai Y, Liu JY, et al. Progress and challenges in the clinical evaluation of immune responses to respiratory mucosal vaccines. Expert Rev Vaccines. 2024;23(1):362–70. 10.1080/14760584.2024.2326094. 38444382 10.1080/14760584.2024.2326094

[39] Ziogas A, Bruno M, van der Meel R, Mulder WJM, Netea MG. Trained immunity: target for prophylaxis and therapy. Cell Host Microbe. 2023;31(11):1776–91. 10.1016/j.chom.2023.10.015. 37944491 10.1016/j.chom.2023.10.015

[40] Netea MG, Domínguez-Andrés J, Barreiro LB, Chavakis T, Divangahi M, Fuchs E, et al. Defining trained immunity and its role in health and disease. Nat Rev Immunol. 2020;20(6):375–88. 10.1038/s41577-020-0285-6. 32132681 10.1038/s41577-020-0285-6 PMC7186935

[41] Chumakov K, Avidan MS, Benn CS, Bertozzi SM, Blatt L, Chang AY et al. Old vaccines for new infections: Exploiting innate immunity to control COVID-19 and prevent future pandemics. P Natl Acad Sci USA. 2021;118(21). ARTN e2101718118. 10.1073/pnas.2101718118. 10.1073/pnas.2101718118 PMC8166166 34006644

[42] Kleinnijenhuis J, Quintin J, Preijers F, Benn CS, Joosten LAB, Jacobs C, et al. Long-lasting effects of BCG vaccination on both heterologous Th1/Th17 responses and innate trained immunity. J Innate Immun. 2014;6(2):152–8. 10.1159/000355628. 24192057 10.1159/000355628 PMC3944069

[43] Lund N, Andersen A, Hansen ASK, Jepsen FS, Barbosa A, Biering-Sorensen S, et al. The Effect of Oral Polio Vaccine at Birth on Infant Mortality: A Randomized Trial. Clin Infect Dis. 2015;61(10):1504–11. 10.1093/cid/civ617. 26219694 10.1093/cid/civ617 PMC4614411

[44] Rieckmann A, Villumsen M, Sorup S, Haugaard LK, Ravn H, Roth A, et al. Vaccinations against smallpox and tuberculosis are associated with better long-term survival: a Danish case-cohort study 1971–2010. Int J Epidemiol. 2017;46(2):695–705. 10.1093/ije/dyw120. 27380797 10.1093/ije/dyw120 PMC5837789

[45] Hong M, Sandalova E, Low D, Gehring AJ, Fieni S, Amadei B et al. Trained immunity in newborn infants of HBV-infected mothers. Nat Commun. 2015;6. ARTN 6588. 10.1038/ncomms7588. 10.1038/ncomms7588 PMC4389241 25807344

[46] Ifrim DC, Quintin J, Joosten LAB, Jacobs C, Jansen T, Jacobs L, et al. Trained immunity or tolerance: opposing functional programs induced in human monocytes after engagement of various pattern recognition receptors. Clin Vaccine Immunol. 2014;21(4):534–45. 10.1128/Cvi.00688-13. 24521784 10.1128/CVI.00688-13 PMC3993125

[47] Guermonprez P, Valladeau J, Zitvogel L, Théry C, Amigorena S. Antigen presentation and T cell stimulation by dendritic cells. Annu Rev Immunol. 2002;20:621–67. 10.1146/annurev.immunol.20.100301.064828. 11861614 10.1146/annurev.immunol.20.100301.064828

[48] Ochando J, Mulder WJM, Madsen JC, Netea MG, Duivenvoorden R. Trained immunity - basic concepts and contributions to immunopathology. Nat Rev Nephrol. 2023;19(1):23–37. 10.1038/s41581-022-00633-5. 36253509 10.1038/s41581-022-00633-5 PMC9575643

[49] Netea MG, Schlitzer A, Placek K, Joosten LAB, Schultze JL. Innate and adaptive immune memory: an evolutionary continuum in the host’s response to pathogens. Cell Host Microbe. 2019;25(1):13–26. 10.1016/j.chom.2018.12.006. 30629914 10.1016/j.chom.2018.12.006

[50] Moorlag S, Rodriguez-Rosales YA, Gillard J, Fanucchi S, Theunissen K, Novakovic B, et al. BCG vaccination induces long-term functional reprogramming of human neutrophils. Cell Rep. 2020;33(7): 108387. 10.1016/j.celrep.2020.108387. 33207187 10.1016/j.celrep.2020.108387 PMC7672522

[51] Amirlak L, Haddad R, Hardy JD, Khaled NS, Chung MH, Amirlak B. Effectiveness of booster BCG vaccination in preventing Covid-19 infection. Hum Vaccin Immunother. 2021;17(11):3913–5. 10.1080/21645515.2021.1956228. 34403297 10.1080/21645515.2021.1956228 PMC8425429

[52] Dotiwala F, Upadhyay AK. Next generation mucosal vaccine strategy for respiratory pathogens. Vaccines. 2023;11(10): ARTN 1585. 10.3390/vaccines11101585. 10.3390/vaccines11101585 PMC10611113 37896988

[53] Tsai CJY, Loh JMS, Fujihashi K, Kiyono H. Mucosal vaccination: onward and upward. Expert Rev Vaccines. 2023;22(1):885–99. 10.1080/14760584.2023.2268724. 37817433 10.1080/14760584.2023.2268724

[54] Zhou X, Wu Y, Zhu Z, Lu C, Zhang C, Zeng L, et al. Mucosal immune response in biology, disease prevention and treatment. Signal Transduct Target Ther. 2025;10(1):7. 10.1038/s41392-024-02043-4. 39774607 10.1038/s41392-024-02043-4 PMC11707400

[55] Kabat AM, Pott J, Maloy KJ. The mucosal immune system and its regulation by autophagy. Front Immunol. 2016;7:240. 10.3389/fimmu.2016.00240. 27446072 10.3389/fimmu.2016.00240 PMC4916208

[56] Mantis NJ, Rol N, Corthesy B. Secretory IgA’s complex roles in immunity and mucosal homeostasis in the gut. Mucosal Immunol. 2011;4(6):603–11. 10.1038/mi.2011.41. 21975936 10.1038/mi.2011.41 PMC3774538

[57] Maeda DLNF, Tian DB, Yu HN, Dar N, Rajasekaran V, Meng S et al. Killed whole-genome reduced-bacteria surface-expressed coronavirus fusion peptide vaccines protect against disease in a porcine model. P Natl Acad Sci USA. 2021;118(18). ARTN e2025622118. 10.1073/pnas.2025622118. 10.1073/pnas.2025622118 PMC8106328 33858942

[58] Shakya AK, Chowdhury MYE, Tao W, Gill HS. Mucosal vaccine delivery: current state and a pediatric perspective. J Control Release. 2016;240:394–413. 10.1016/j.jconrel.2016.02.014. 26860287 10.1016/j.jconrel.2016.02.014 PMC5381653

[59] Carreto-Binaghi LE, Sztein MB, Booth JS. Role of cellular effectors in the induction and maintenance of IgA responses leading to protective immunity against enteric bacterial pathogens. Front Immunol. 2024;15:1446072. 10.3389/fimmu.2024.1446072. 39324143 10.3389/fimmu.2024.1446072 PMC11422102

[60] Cheng Q, Khodadadi L, Taddeo A, Klotsche J, B FH, Radbruch A, Hiepe F. CXCR4-CXCL12 interaction is important for plasma cell homing and survival in NZB/W mice. Eur J Immunol. 2018;48(6):1020–9. 10.1002/eji.201747023. 10.1002/eji.201747023 29427452

[61] Zhao M, Zhou L, Wang S. Immune crosstalk between respiratory and intestinal mucosal tissues in respiratory infections. Mucosal Immunol. 2025;18(3):501–8. 10.1016/j.mucimm.2024.12.013. 39755173 10.1016/j.mucimm.2024.12.013

[62] Pabst O. New concepts in the generation and functions of IgA. Nat Rev Immunol. 2012;12(12):821–32. 10.1038/nri3322. 23103985 10.1038/nri3322

[63] Kubinak JL, Petersen C, Stephens WZ, Soto R, Bake E, O’Connell RM, Round JL. MyD88 signaling in T cells directs IgA-mediated control of the microbiota to promote health. Cell Host Microbe. 2015;17(2):153–63. 10.1016/j.chom.2014.12.009. 25620548 10.1016/j.chom.2014.12.009 PMC4451207

[64] Boyaka PN. Inducing mucosal IgA: a challenge for vaccine adjuvants and delivery systems. J Immunol. 2017;199(1):9–16. 10.4049/jimmunol.1601775. 28630108 10.4049/jimmunol.1601775 PMC5719502

[65] Cerutti A. The regulation of IgA class switching. Nat Rev Immunol. 2008;8(6):421–34. 10.1038/nri2322. 18483500 10.1038/nri2322 PMC3062538

[66] Tejedor Vaquero S, Neuman H, Comerma L, Marcos-Fa X, Corral-Vazquez C, Uzzan M, et al. Immunomolecular and reactivity landscapes of gut IgA subclasses in homeostasis and inflammatory bowel disease. J Exp Med. 2024. 10.1084/jem.20230079. 10.1084/jem.20230079 PMC11577441 39560666

[67] Scheurer S, Junker AC, He C, Schulke S, Toda M. The role of IgA in the manifestation and prevention of allergic immune responses. Curr Allergy Asthma Rep. 2023;23(10):589–600. 10.1007/s11882-023-01105-x. 37610671 10.1007/s11882-023-01105-x PMC10506939

[68] Rojas R, Apodaca G. Immunoglobulin transport across polarized epithelial cells. Nat Rev Mol Cell Biol. 2002;3(12):944–55. 10.1038/nrm972. 12461560 10.1038/nrm972

[69] Woof JM, Russell MW. Structure and function relationships in IgA. Mucosal Immunol. 2011;4(6):590–7. 10.1038/mi.2011.39. 21937984 10.1038/mi.2011.39

[70] Suzuki T, Kawaguchi A, Ainai A, Tamura S, Ito R, Multihartina P, et al. Relationship of the quaternary structure of human secretory IgA to neutralization of influenza virus. P Natl Acad Sci USA. 2015;112(25):7809–14. 10.1073/pnas.1503885112. 10.1073/pnas.1503885112 PMC4485102 26056267

[71] Renegar KB, Small PA, Boykins LG, Wright PF. Role of IgA versus IgG in the control of influenza viral infection in the murine respiratory tract. J Immunol. 2004;173(3):1978–86. 10.4049/jimmunol.173.3.1978. 15265932 10.4049/jimmunol.173.3.1978

[72] Brokstad KA, Cox RJ, Eriksson JC, Olofsson J, Jonsson R, Davidsson A. High prevalence of influenza specific antibody secreting cells in nasal mucosa. Scand J Immunol. 2001;54(1–2):243–7. 10.1046/j.1365-3083.2001.00947.x. 11439173 10.1046/j.1365-3083.2001.00947.x

[73] Marking U, Bladh O, Havervall S, Svensson J, Greilert-Norin N, Aguilera K, et al. 7-month duration of SARS-CoV-2 mucosal immunoglobulin-a responses and protection. Lancet Infect Dis. 2023;23(2):150–2. 10.1016/S1473-3099(22)00834-9. 36640796 10.1016/S1473-3099(22)00834-9 PMC9833832

[74] Zuo FL, Marcotte H, Hammarström L, Pan-Hammarström Q. Mucosal IgA against SARS-CoV-2 Omicron Infection. New England Journal of Medicine. 2022;387(21). ARTN e55. 10.1056/NEJMc2213153. 10.1056/NEJMc2213153 36416778

[75] Sheikh-Mohamed S, Isho B, Chao GYC, Zuo M, Cohen C, Lustig Y, et al. Systemic and mucosal IgA responses are variably induced in response to SARS-CoV-2 mRNA vaccination and are associated with protection against subsequent infection. Mucosal Immunol. 2022. 10.1038/s41385-022-00511-0. 35468942 10.1038/s41385-022-00511-0 PMC9037584

[76] Miyamoto S, Nishiyama T, Ueno A, Park H, Kanno T, Nakamura N et al. Infectious virus shedding duration reflects secretory IgA antibody response latency after SARS-CoV-2 infection. P Natl Acad Sci USA. 2023;120(52). ARTN e2314808120. 10.1073/pnas.2314808120. 10.1073/pnas.2314808120 PMC10756199 38134196

[77] Kwon DI, Mao T, Israelow B, Santos Guedes de Sa K, Dong H, Iwasaki A. Mucosal unadjuvanted booster vaccines elicit local IgA responses by conversion of pre-existing immunity in mice. Nat Immunol. 2025;26(6):908–19. 10.1038/s41590-025-02156-0. 40360777 10.1038/s41590-025-02156-0 PMC12133566

[78] Worzner K, Schmidt ST, Zimmermann J, Tami A, Polacek C, Fernandez-Antunez C, et al. Intranasal recombinant protein subunit vaccine targeting TLR3 induces respiratory tract IgA and CD8 T cell responses and protects against respiratory virus infection. EBioMedicine. 2025;113: 105615. 10.1016/j.ebiom.2025.105615. 39983329 10.1016/j.ebiom.2025.105615 PMC11893338

[79] Liu Y, Lam DM, Luan M, Zheng W, Ai H. Recent development of oral vaccines (review). Exp Ther Med. 2024;27(5): 223. 10.3892/etm.2024.12511. 38590568 10.3892/etm.2024.12511 PMC11000446

[80] Pilapitiya D, Wheatley AK, Tan HX. Mucosal vaccines for SARS-CoV-2: triumph of hope over experience. EBioMedicine. 2023;92: 104585. 10.1016/j.ebiom.2023.104585. 37146404 10.1016/j.ebiom.2023.104585 PMC10154910

[81] Mackay LK, Rahimpour A, Ma JZ, Collins N, Stock AT, Hafon ML, et al. The developmental pathway for CD103CD8 tissue-resident memory T cells of skin. Nat Immunol. 2013;14(12):1294. 10.1038/ni.2744. 24162776 10.1038/ni.2744

[82] Shiow LR, Rosen DB, Brdicková N, Xu Y, An JP, Lanier LL, et al. CD69 acts downstream of interferon-α/β to inhibit S1P and lymphocyte egress from lymphoid organs. Nature. 2006;440(7083):540–4. 10.1038/nature04606. 16525420 10.1038/nature04606

[83] Szabo PA, Miron M, Farber DL. Location, location, location: Tissue resident memory T cells in mice and humans. Sci Immunol. 2019;4(34). ARTN eaas9673. 10.1126/sciimmunol.aas9673. 10.1126/sciimmunol.aas9673 PMC6778482 30952804

[84] Wein AN, McMaster SR, Takamura S, Dunbar PR, Cartwright EK, Hayward SL, et al. CXCR6 regulates localization of tissue-resident memory CD8 T cells to the airways. J Exp Med. 2019;216(12):2748–62. 10.1084/jem.20181308. 31558615 10.1084/jem.20181308 PMC6888981

[85] Hombrink P, Helbig C, Backer RA, Piet B, Oja AE, Stark R, et al. Programs for the persistence, vigilance and control of human CD8^+^ lung-resident memory T cells. Nat Immunol. 2016;17(12):1467–78. 10.1038/ni.3589. 27776108 10.1038/ni.3589

[86] Jozwik A, Habibi MS, Paras A, Zhu J, Guvenel A, Dhariwal J et al. RSV-specific airway resident memory CD8+T cells and differential disease severity after experimental human infection. Nat Commun. 2015;6. ARTN 10224. 10.1038/ncomms10224. 10.1038/ncomms10224 PMC4703893 26687547

[87] Laidlaw BJ, Ellebedy AH. The germinal centre B cell response to SARS-CoV-2. Nat Rev Immunol. 2022;22(1):7–18. 10.1038/s41577-021-00657-1. 34873279 10.1038/s41577-021-00657-1 PMC8647067

[88] Bartolomé-Casado R, Landsverk OJB, Chauhan SK, Sætre F, Hagen KT, Yaqub S, et al. CD4^+^T cells persist for years in the human small intestine and display a T1 cytokine profile. Mucosal Immunol. 2021;14(2):402–10. 10.1038/s41385-020-0315-5. 32572129 10.1038/s41385-020-0315-5

[89] Schreiner D, King CG. CD4+Memory T Cells at Home in the Tissue: Mechanisms for Health and Disease. Frontiers in Immunology. 2018;9. ARTN 2394. 10.3389/fimmu.2018.02394. 10.3389/fimmu.2018.02394 PMC6198086 30386342

[90] Teijaro JR, Turner D, Pham Q, Wherry EJ, Lefrancois L, Farber DL. Cutting edge: Tissue-retentive lung memory CD4 T cells mediate optimal protection to respiratory virus infection. J Immunol. 2011;187(11):5510–4. 10.4049/jimmunol.1102243. 22058417 10.4049/jimmunol.1102243 PMC3221837

[91] Laidlaw BJ, Zhang N, Marshall HD, Staron MM, Guan T, Hu Y, et al. CD4+ t cell help guides formation of CD103+ lung-resident memory CD8+ t cells during influenza viral infection. Immunity. 2014;41(4):633–45. 10.1016/j.immuni.2014.09.007. 25308332 10.1016/j.immuni.2014.09.007 PMC4324721

[92] O’Hara JM, Redhu NS, Cheung E, Robertson NG, Patik I, El Sayed S, et al. Generation of protective pneumococcal-specific nasal resident memory CD4 T cells via parenteral immunization. Mucosal Immunol. 2020;13(1):172–82. 10.1038/s41385-019-0218-5. 31659300 10.1038/s41385-019-0218-5 PMC6917870

[93] Zens KD, Chen JK, Farber DL. Vaccine-generated lung tissue-resident memory T cells provide heterosubtypic protection to influenza infection. Jci Insight. 2016;1(10). ARTN e85832. 10.1172/jci.insight.85832. 10.1172/jci.insight.85832 PMC4959801 27468427

[94] Slütter B, Van Braeckel-Budimir N, Abboud G, Varga SM, Salek-Ardakani S, Harty JT. Dynamics of influenza-induced lung-resident memory T cells underlie waning heterosubtypic immunity. Sci Immunol. 2017;2(7). ARTN eaag2031. 10.1126/sciimmunol.aag2031. 28783666 10.1126/sciimmunol.aag2031 PMC5590757

[95] Uddbäck I, Cartwright EK, Scholler AS, Wein AN, Hayward SL, Lobby J, et al. Long-term maintenance of lung resident memory T cells is mediated by persistent antigen. Mucosal Immunol. 2021;14(1):92–9. 10.1038/s41385-020-0309-3. 32518368 10.1038/s41385-020-0309-3 PMC7726002

[96] Barker KA, Etesami NS, Shenoy AT, Arafa EI, Lyon de Ana C, Smith NM et al. Lung-resident memory B cells protect against bacterial pneumonia. J Clin Invest. 2021;131(11). 10.1172/JCI141810. 10.1172/JCI141810 PMC8159694 34060477

[97] Oh JE, Song E, Moriyama M, Wong P, Zhang S, Jiang RY et al. Intranasal priming induces local lung-resident B cell populations that secrete protective mucosal antiviral IgA. Sci Immunol. 2021;6(66):abj5129. 10.1126/sciimmunol.abj5129. 10.1126/sciimmunol.abj5129 PMC8762609 34890255

[98] Tan HX, Esterbauer R, Vanderven HA, Juno JA, Kent SJ, Wheatley AK. Inducible Bronchus-Associated Lymphoid Tissues (iBALT) Serve as Sites of B Cell Selection and Maturation Following Influenza Infection in Mice. Frontiers in Immunology. 2019;10:611. 10.3389/fimmu.2019.00611. 30984186 10.3389/fimmu.2019.00611 PMC6450362

[99] Wang MY, Zhao R, Gao LJ, Gao XF, Wang DP, Cao JM. SARS-CoV-2: Structure, Biology, and Structure-Based Therapeutics Development. Front Cell Infect Mi. 2020;10:587269. 10.3389/fcimb.2020.587269. 10.3389/fcimb.2020.587269 PMC7723891 33324574

[100] MacLean AJ, Richmond N, Koneva L, Attar M, Medina CAP, Thornton EE, et al. Secondary influenza challenge triggers resident memory B cell migration and rapid relocation to boost antibody secretion at infected sites. Immunity. 2022;55(4):718. 10.1016/j.immuni.2022.03.003. 35349789 10.1016/j.immuni.2022.03.003 PMC9044924

[101] Onodera T, Takahashi Y, Yokoi Y, Ato M, Kodama Y, Hachimura S, et al. Memory B cells in the lung participate in protective humoral immune responses to pulmonary influenza virus reinfection. P Natl Acad Sci USA. 2012;109(7):2485–90. 10.1073/pnas.1115369109. 10.1073/pnas.1115369109 PMC3289300 22308386

[102] Gregoire C, Spinelli L, Villazala-Merino S, Gil L, Holgado MP, Moussa M, et al. Viral infection engenders bona fide and bystander subsets of lung-resident memory B cells through a permissive mechanism. Immunity. 2022;55(7):1216. 10.1016/j.immuni.2022.06.002. 35768001 10.1016/j.immuni.2022.06.002 PMC9396418

[103] Whitsett JA. Airway epithelial differentiation and mucociliary clearance. Ann Am Thorac Soc. 2018;15:S143–8. 10.1513/AnnalsATS.201802-128AW. 30431340 10.1513/AnnalsATS.201802-128AW PMC6322033

[104] Chatterjee M, van Putten JPM, Strijbis K. Defensive Properties of Mucin Glycoproteins during Respiratory Infections-Relevance for SARS-CoV-2. Mbio. 2020;11(6):e02374–20. 10.1128/mBio.02374-20. 10.1128/mBio.02374-20 PMC7663010 33184103

[105] Liang B, Xing DM. Unveiling the mystery of ILC3s: Their functions and interactions in mucosal immunity. Int Immunopharmacol. 2023;123:110772. 10.1016/j.intimp.2023.110772. 37552906 10.1016/j.intimp.2023.110772

[106] Simoni Y, Fehlings M, Kloverpris HN, McGovern N, Koo SL, Loh CY, et al. Human Innate Lymphoid Cell Subsets Possess Tissue-Type Based Heterogeneity in Phenotype and Frequency. Immunity. 2017;46(1):148–61. 10.1016/j.immuni.2016.11.005. 27986455 10.1016/j.immuni.2016.11.005 PMC7612935

[107] Mirchandani AS, Besnard AG, Yip E, Scott C, Bain CC, Cerovic V, et al. Type 2 innate lymphoid cells drive CD4 Th2 cell responses. J Immunol. 2014;192(5):2442–8. 10.4049/jimmunol.1300974. 24470502 10.4049/jimmunol.1300974

[108] Neill DR, Wong SH, Bellosi A, Flynn RJ, Daly M, Langford TKA, et al. Nuocytes represent a new innate effector leukocyte that mediates type-2 immunity. Nature. 2010;464(7293):1367-U9. 10.1038/nature08900. 20200518 10.1038/nature08900 PMC2862165

[109] Ardain A, Porterfield JZ, Kloverpris HN, Leslie A. Type 3 ILCs in Lung Disease. Frontiers in Immunology. 2019;10. ARTN 92. 10.3389/fimmu.2019.00092. 10.3389/fimmu.2019.00092 PMC6361816 30761149

[110] Yu XY, Buttgereit A, Lelios I, Utz SG, Cansever D, Becher B, Greter M. The cytokine TGF-β promotes the development and homeostasis of alveolar macrophages. Immunity. 2017;47(5):903. 10.1016/j.immuni.2017.10.007. 29126797 10.1016/j.immuni.2017.10.007

[111] Harabuchi Y, Takahara M. Recent advances in the immunological understanding of association between tonsil and immunoglobulin A nephropathy as a tonsil-induced autoimmune/inflammatory syndrome. Immun Inflamm Dis. 2019;7(2):86–92. 10.1002/iid3.248. 30957421 10.1002/iid3.248 PMC6485698

[112] Gesualdo L, Di Leo V, Coppo R. The mucosal immune system and IgA nephropathy. Semin Immunopathol. 2021;43(5):657–68. 10.1007/s00281-021-00871-y. 34642783 10.1007/s00281-021-00871-y PMC8551125

[113] Teng Z, Meng LY, Yang JK, He Z, Chen XG, Liu Y. Bridging nanoplatform and vaccine delivery, a landscape of strategy to enhance nasal immunity. J Control Release. 2022;351:456–75. 10.1016/j.jconrel.2022.09.044. 36174803 10.1016/j.jconrel.2022.09.044

[114] Nakahashi-Ouchida R, Fujihashi K, Kurashima Y, Yuki Y, Kiyono H. Nasal vaccines: solutions for respiratory infectious diseases. Trends Mol Med. 2023;29(2):124–40. 10.1016/j.molmed.2022.10.009. 36435633 10.1016/j.molmed.2022.10.009

[115] Christensen D, Mortensen R, Rosenkrands I, Dietrich J, Andersen P. Vaccine-induced Th17 cells are established as resident memory cells in the lung and promote local IgA responses. Mucosal Immunol. 2017;10(1):260–70. 10.1038/mi.2016.28. 27049058 10.1038/mi.2016.28

[116] Nigwekar PV, Kumar A, Padbidri VV, Choudhury A, Chaudhari AB, Kulkarni PS. Safety of Russian-Backbone trivalent, live attenuated seasonal influenza vaccine in healthy subjects: open-label, non-randomized phase 4 study. Drug Saf. 2018;41(2):171–7. 10.1007/s40264-017-0605-3. 29027148 10.1007/s40264-017-0605-3

[117] Bull NC, Stylianou E, Kaveh DA, Pinpathomrat N, Pasricha J, Harrington-Kandt R, et al. Enhanced protection conferred by mucosal BCG vaccination associates with presence of antigen-specific lung tissue-resident PD-1(+) KLRG1(-) CD4(+) T cells. Mucosal Immunol. 2019;12(2):555–64. 10.1038/s41385-018-0109-1. 30446726 10.1038/s41385-018-0109-1 PMC7051908

[118] Keech C, Miller VE, Rizzardi B, Hoyle C, Pryor MJ, Ferrand J, et al. Immunogenicity and safety of BPZE1, an intranasal live attenuated pertussis vaccine, versus tetanus-diphtheria-acellular pertussis vaccine: a randomised, double-blind, phase 2b trial. Lancet. 2023;401(10379):843–55. 10.1016/S0140-6736(22)02644-7. 36906345 10.1016/S0140-6736(22)02644-7

[119] Jahnmatz M, Richert L, Al-Tawil N, Storsaeter J, Colin C, Bauduin C, et al. Safety and immunogenicity of the live attenuated intranasal pertussis vaccine BPZE1: a phase 1b, double-blind, randomised, placebo-controlled dose-escalation study. Lancet Infect Dis. 2020;20(11):1290–301. 10.1016/S1473-3099(20)30274-7. 32687804 10.1016/S1473-3099(20)30274-7

[120] Chavda VP, Baviskar KP, Vaghela DA, Raut SS, Bedse AP. Nasal sprays for treating COVID-19: a scientific note. Pharmacol Rep. 2023;75(2):249–65. 10.1007/s43440-023-00463-7. 36848033 10.1007/s43440-023-00463-7 PMC9969373

[121] Davitt CJH, McNeela EA, Longet S, Tobias J, Aversa V, McEntee CP, et al. A novel adjuvanted capsule based strategy for oral vaccination against infectious diarrhoeal pathogens. J Control Release. 2016;233:162–73. 10.1016/j.jconrel.2016.05.001. 27157995 10.1016/j.jconrel.2016.05.001

[122] Connor RI, Brickley EB, Wieland-Alter WF, Ackerman ME, Weiner JA, Modlin JF, et al. Mucosal immunity to poliovirus. Mucosal Immunol. 2022;15(1):1–9. 10.1038/s41385-021-00428-0. 34239028 10.1038/s41385-021-00428-0 PMC8732262

[123] Morbe UM, Jorgensen PB, Fenton TM, von Burg N, Riis LB, Spencer J, Agace WW. Human gut-associated lymphoid tissues (GALT); diversity, structure, and function. Mucosal Immunol. 2021;14(4):793–802. 10.1038/s41385-021-00389-4. 33753873 10.1038/s41385-021-00389-4

[124] Li GQ, Xia J, Zeng W, Luo W, Liu L, Zeng X, Cao D. The intestinal gammadelta T cells: functions in the gut and in the distant organs. Front Immunol. 2023;14:1206299. 10.3389/fimmu.2023.1206299. 37398661 10.3389/fimmu.2023.1206299 PMC10311558

[125] Lockhart A, Mucida D, Parsa R. Immunity to enteric viruses. Immunity. 2022;55(5):800–18. 10.1016/j.immuni.2022.04.007. 35545029 10.1016/j.immuni.2022.04.007 PMC9257994

[126] Ciabattini A, Pettini E, Arsenijevic S, Pozzi G, Medaglini D. Intranasal immunization with vaccine vector elicits primed CD4 and CD8 T cells in the genital and intestinal tracts. Vaccine. 2010;28(5):1226–33. 10.1016/j.vaccine.2009.11.021. 19945415 10.1016/j.vaccine.2009.11.021

[127] Vela Ramirez JE, Sharpe LA, Peppas NA. Current state and challenges in developing oral vaccines. Adv Drug Deliv Rev. 2017;114:116–31. 10.1016/j.addr.2017.04.008. 28438674 10.1016/j.addr.2017.04.008 PMC6132247

[128] Marsland BJ, Trompette A, Gollwitzer ES. The Gut-Lung Axis in Respiratory Disease. Ann Am Thorac Soc. 2015;12:S150–6. 10.1513/AnnalsATS.201503-133AW. 26595731 10.1513/AnnalsATS.201503-133AW

[129] Giannelli V, Di Gregorio V, Iebba V, Giusto M, Schippa S, Merli M, Thalheimer U. Microbiota and the gut-liver axis: Bacterial translocation, inflammation and infection in cirrhosis. World J Gastroentero. 2014;20(45):16795–810. 10.3748/wjg.v20.i45.16795. 10.3748/wjg.v20.i45.16795 PMC4258550 25492994

[130] Yang T, Richards EM, Pepine CJ, Raizada MK. The gut microbiota and the brain-gut-kidney axis in hypertension and chronic kidney disease. Nat Rev Nephrol. 2018;14(7):442–56. 10.1038/s41581-018-0018-2. 29760448 10.1038/s41581-018-0018-2 PMC6385605

[131] Lei YMK, Nair L, Alegre ML. The interplay between the intestinal microbiota and the immune system. Clin Res Hepatol Gas. 2015;39(1):9–19. 10.1016/j.clinre.2014.10.008. 10.1016/j.clinre.2014.10.008 PMC4423786 25481240

[132] Thaiss CA, Levy M, Suez J, Elinav E. The interplay between the innate immune system and the microbiota. Curr Opin Immunol. 2014;26:41–8. 10.1016/j.coi.2013.10.016. 24556399 10.1016/j.coi.2013.10.016

[133] Chattha KS, Roth JA, Saif LJ. Strategies for design and application of enteric viral vaccines. Annu Rev Anim Biosci. 2015;3:375–95. 10.1146/annurev-animal-022114-111038. 25387111 10.1146/annurev-animal-022114-111038

[134] Holscher HD, Czerkies LA, Cekola P, Litov R, Benbow M, Santema S, et al. Bb12 Enhances Intestinal Antibody Response in Formula-Fed Infants: A Randomized, Double-Blind. Controlled Trial Jpen-Parenter Enter. 2012;36:106s–17s. 10.1177/0148607111430817. 10.1177/0148607111430817 22237870

[135] Kayama H, Takeda K. Functions of innate immune cells and commensal bacteria in gut homeostasis. J Biochem. 2016;159(2):141–9. 10.1093/jb/mvv119. 26615026 10.1093/jb/mvv119 PMC4892783

[136] Lindqvist M, Persson J, Thorn K, Harandi AM. The Mucosal Adjuvant Effect of α-Galactosylceramide for Induction of Protective Immunity to Sexually Transmitted Viral Infection. J Immunol. 2009;182(10):6435–43. 10.4049/jimmunol.0900136. 19414797 10.4049/jimmunol.0900136

[137] Oh JE, Iijima N, Song E, Lu P, Klein J, Jiang R, et al. Migrant memory B cells secrete luminal antibody in the vagina. Nature. 2019;571(7763):122–6. 10.1038/s41586-019-1285-1. 31189952 10.1038/s41586-019-1285-1 PMC6609483

[138] Tan HX, Wheatley AK, Esterbauer R, Jegaskanda S, Glass JJ, Masopust D, et al. Induction of vaginal-resident HIV-specific CD8 T cells with mucosal prime-boost immunization. Mucosal Immunol. 2018;11(3):994–1007. 10.1038/mi.2017.89. 29067995 10.1038/mi.2017.89

[139] Mokabari K, Iriti M, Varoni EM. Mucoadhesive vaccine delivery systems for the oral mucosa. J Dent Res. 2023;102(7):709–18. 10.1177/00220345231164111. 37148290 10.1177/00220345231164111

[140] Oya Y, Kimura S, Nakamura Y, Ishihara N, Takano S, Morita R et al. Characterization of M Cells in Tear Duct-Associated Lymphoid Tissue of Mice: A Potential Role in Immunosurveillance on the Ocular Surface. Frontiers in Immunology. 2021;12:779709. 10.3389/fimmu.2021.779709. 34880872 10.3389/fimmu.2021.779709 PMC8645900

[141] Kim J, Kim ED, Shin HS, Han SJ, Jamiyansharav M, Yoon SC, et al. Effectiveness and safety of injectable human papilloma virus vaccine administered as eyedrops. Vaccine. 2023;41(1):92–100. 10.1016/j.vaccine.2022.09.070. 36402660 10.1016/j.vaccine.2022.09.070

[142] Seo KY, Han SJ, Cha HR, Seo SU, Song JH, Chung SH, Kweon MN. Eye mucosa: an efficient vaccine delivery route for inducing protective immunity. J Immunol. 2010;185(6):3610–9. 10.4049/jimmunol.1000680. 20709955 10.4049/jimmunol.1000680

[143] Thakur A, Foged C. Nanoparticles for mucosal vaccine delivery. Nanoengineered Biomaterials for Advanced Drug Delivery. 2020. p. 603–46.

[144] Sahay B, Colliou N, Zadeh M, Ge Y, Gong MH, Owen JL, et al. Dual-route targeted vaccine protects efficiently against botulinum neurotoxin A complex. Vaccine. 2018;36(1):155–64. 10.1016/j.vaccine.2017.11.008. 29180028 10.1016/j.vaccine.2017.11.008 PMC6180320

[145] Stary G, Olive A, Radovic-Moreno AF, Gondek D, Alvarez D, Basto PA et al. A mucosal vaccine against generates two waves of protective memory T cells. Science. 2015;348(6241):aaa8205. 10.1126/science.aaa8205. 26089520 10.1126/science.aaa8205 PMC4605428

[146] Moyle PM, Toth I. Modern subunit vaccines: development, components, and research opportunities. ChemMedChem. 2013;8(3):360–76. 10.1002/cmdc.201200487. 23316023 10.1002/cmdc.201200487

[147] Li YD, Chi WY, Su JH, Ferrall L, Hung CF, Wu TC. Coronavirus vaccine development: from SARS and MERS to COVID-19. J Biomed Sci. 2020;27(1):104. 10.1186/s12929-020-00695-2. 33341119 10.1186/s12929-020-00695-2 PMC7749790

[148] Minor PD. Live attenuated vaccines: historical successes and current challenges. Virology. 2015;479:379–92. 10.1016/j.virol.2015.03.032. 25864107 10.1016/j.virol.2015.03.032

[149] Reed SG, Orr MT, Fox CB. Key roles of adjuvants in modern vaccines. Nat Med. 2013;19(12):1597–608. 10.1038/nm.3409. 24309663 10.1038/nm.3409

[150] Jeyanathan M, Afkhami S, Smaill F, Miller MS, Lichty BD, Xing Z. Immunological considerations for COVID-19 vaccine strategies. Nat Rev Immunol. 2020;20(10):615–32. 10.1038/s41577-020-00434-6. 32887954 10.1038/s41577-020-00434-6 PMC7472682

[151] Schmidt A, Lapuente D. T cell immunity against influenza: the long way from animal models towards a real-life universal flu vaccine. Viruses. 2021;13(2): ARTN 199. 10.3390/v13020199. 10.3390/v13020199 PMC7911237 33525620

[152] Wherry EJ, Barouch DH. T cell immunity to COVID-19 vaccines. Science. 2022;377(6608):821–2. 10.1126/science.add2897. 35981045 10.1126/science.add2897

[153] Travieso T, Li J, Mahesh S, Mello JDRE, Blasi M. The use of viral vectors in vaccine development. Npj Vaccines. 2022;7(1):75. 10.1038/s41541-022-00503-y. 35787629 10.1038/s41541-022-00503-y PMC9253346

[154] Toniolo A, Maccari G, Camussi G. mRNA technology and mucosal immunization. Vaccines. 2024;12(6): ARTN 670. 10.3390/vaccines12060670. 10.3390/vaccines12060670 PMC11209623 38932399

[155] Hameed SA, Paul S, Dellosa GKY, Jaraquemada D, Bello MB. Towards the future exploration of mucosal mRNA vaccines against emerging viral diseases; lessons from existing next-generation mucosal vaccine strategies. Npj Vaccines. 2022;7(1). ARTN 71. 10.1038/s41541-022-00485-x. 10.1038/s41541-022-00485-x PMC9239993 35764661

[156] Song YF, Mehl F, Zeichner SL. Vaccine strategies to elicit mucosal immunity. Vaccines. 2024;12(2): ARTN 191. 10.3390/vaccines12020191. 10.3390/vaccines12020191 PMC10892965 38400174

[157] Yeung J, Wang T, Shi PY. Improvement of mucosal immunity by a live-attenuated SARS-CoV-2 nasal vaccine. Curr Opin Virol. 2023;62:101347. 10.1016/j.coviro.2023.101347. 37604085 10.1016/j.coviro.2023.101347

[158] Luquero FJ, Grout L, Ciglenecki I, Sakoba K, Traore B, Heile M et al. First Outbreak Response Using an Oral Cholera Vaccine in Africa: Vaccine Coverage, Acceptability and Surveillance of Adverse Events, Guinea, 2012. Plos Neglect Trop D. 2013;7(10):e2465. 10.1371/journal.pntd.0002465. 10.1371/journal.pntd.0002465 PMC3798604 24147164

[159] Mosley JF 2nd, Smith LL, Brantley P, Locke D, Como M. Vaxchora: The First FDA-Approved Cholera Vaccination in the United States. P T. 2017;42(10):638–40. 29018300 PMC5614415

[160] Platt LR, Estívariz CF, Sutter RW. Vaccine-Associated Paralytic Poliomyelitis: A Review of the Epidemiology and Estimation of the Global Burden. J Infect Dis. 2014;210:S380–9. 10.1093/infdis/jiu184. 25316859 10.1093/infdis/jiu184 PMC10424844

[161] Xu H, Cai L, Hufnagel S, Cui Z. Intranasal vaccine: factors to consider in research and development. Int J Pharm. 2021;609: 121180. 10.1016/j.ijpharm.2021.121180. 34637935 10.1016/j.ijpharm.2021.121180

[162] Verma SK, Mahajan P, Singh NK, Gupta A, Aggarwal R, Rappuoli R, Johri AK. New-age vaccine adjuvants, their development, and future perspective. Front Immunol. 2023;14:1043109. 10.3389/fimmu.2023.1043109. 36911719 10.3389/fimmu.2023.1043109 PMC9998920

[163] Terrinoni M, Nordqvist SL, Löfstrand M, Nilsson F, Källgård S, Sharma T, et al. A thermostable, dry formulation inactivated Hikojima whole cell/cholera toxin B subunit oral cholera vaccine. Vaccine. 2023;41(21):3347–57. 10.1016/j.vaccine.2023.04.004. 37085452 10.1016/j.vaccine.2023.04.004

[164] Azeem M, Cancemi P, Mukhtar F, Marino S, Peri E, Di Prima G, De Caro V. Efficacy and limitations of SARS-CoV-2 vaccines - A systematic review. Life Sci. 2025;371: 123610. 10.1016/j.lfs.2025.123610. 40189198 10.1016/j.lfs.2025.123610

[165] Krammer F, Palese P. Universal influenza virus vaccines that target the conserved hemagglutinin stalk and conserved sites in the head domain. J Infect Dis. 2019;219:S62–7. 10.1093/infdis/jiy711. 30715353 10.1093/infdis/jiy711 PMC6452318

[166] Edgar JE, Trezise S, Anthony RM, Krammer F, Palese P, Ravetch JV, Bournazos S. Antibodies elicited in humans upon chimeric hemagglutinin-based influenza virus vaccination confer FcγR-dependent protection in vivo. P Natl Acad Sci USA. 2023;120(44):e2314905120. 10.1073/pnas.2314905120. 10.1073/pnas.2314905120 PMC10622865 37871218

[167] Isakova-Sivak I, Korenkov D, Smolonogina T, Kotomina T, Donina S, Matyushenko V, et al. Broadly protective anti-hemagglutinin stalk antibodies induced by live attenuated influenza vaccine expressing chimeric hemagglutinin. Virology. 2018;518:313–23. 10.1016/j.virol.2018.03.013. 29574336 10.1016/j.virol.2018.03.013

[168] Parish LA, Rele S, Hofmeyer KA, Luck BB, Wolfe DN. Strategic and technical considerations in manufacturing viral vector vaccines for the Biomedical Advanced Research and Development Authority threats. Vaccines. 2025. 10.3390/vaccines13010073. 39852852 10.3390/vaccines13010073 PMC11769106

[169] Sandbrink JB, Koblentz GD. Biosecurity risks associated with vaccine platform technologies. Vaccine. 2022;40(17):2514–23. 10.1016/j.vaccine.2021.02.023. 33640142 10.1016/j.vaccine.2021.02.023 PMC7904460

[170] Rollier CS, Reyes-Sandoval A, Cottingham MG, Ewer K, Hill AVS. Viral vectors as vaccine platforms: deployment in sight. Curr Opin Immunol. 2011;23(3):377–82. 10.1016/j.coi.2011.03.006. 21514130 10.1016/j.coi.2011.03.006

[171] Fallahi MJ, Shahri NE, Khodamoradi Z, Nia MM, Sehatpour F, Mahmoudi L. Case of possible encephalopathy following receiving the first dose of Iranian COVID-19Vaccine; COVIran Barakat. Clin Case Rep. 2022;10(4). ARTN e05661. 10.1002/ccr3.5661. 10.1002/ccr3.5661 PMC8989018 35425597

[172] Yuki Y, Nojima M, Kashima K, Sugiura K, Maruyama S, Kurokawa S, et al. Oral MucoRice-CTB vaccine is safe and immunogenic in healthy US adults. Vaccine. 2022;40(24):3372–9. 10.1016/j.vaccine.2022.04.051. 35484039 10.1016/j.vaccine.2022.04.051

[173] Garg NK, Mangal S, Khambete H, Tyagi RK. Mucosal delivery of vaccines: role of mucoadhesive/biodegradable polymers. Recent Pat Drug Deliv Formul. 2010;4(2):114–28. 10.2174/187221110791185015. 20380624 10.2174/187221110791185015

[174] Nakahashi-Ouchida R, Yuki Y, Kiyono H. Cationic pullulan nanogel as a safe and effective nasal vaccine delivery system for respiratory infectious diseases. Hum Vacc Immunother. 2018;14(9):2189–93. 10.1080/21645515.2018.1461298. 10.1080/21645515.2018.1461298 PMC6183202 29624474

[175] Kyriakidis NC, López-Cortés A, González EV, Grimaldos AB, Prado EO. SARS-CoV-2 vaccines strategies: a comprehensive review of phase 3 candidates. Npj Vaccines. 2021;6(1). ARTN 28. 10.1038/s41541-021-00292-w. 10.1038/s41541-021-00292-w PMC7900244 33619260

[176] Park JH, Lee HK. Delivery routes for COVID-19 vaccines. Vaccines. 2021;9(5): ARTN 524. 10.3390/vaccines9050524. 10.3390/vaccines9050524 PMC8158705 34069359

[177] Wang S, Liang B, Wang WQ, Li L, Feng N, Zhao YK et al. Viral vectored vaccines: design, development, preventive and therapeutic applications in human diseases. Signal Transduct Tar. 2023;8(1):149. 10.1038/s41392-023-01408-5. 10.1038/s41392-023-01408-5 PMC10081433 37029123

[178] Jeyanathan M, Afkhami S, Kang A, Xing Z. Viral-vectored respiratory mucosal vaccine strategies. Curr Opin Immunol. 2023;84:102370. 10.1016/j.coi.2023.102370. 37499279 10.1016/j.coi.2023.102370

[179] Satti I, Meyer J, Harris SA, Thomas ZRM, Griffiths K, Antrobus RD, et al. Safety and immunogenicity of a candidate tuberculosis vaccine MVA85A delivered by aerosol in BCG-vaccinated healthy adults: a phase 1, double-blind, randomised controlled trial. Lancet Infect Dis. 2014;14(10):939–46. 10.1016/S1473-3099(14)70845-X. 25151225 10.1016/S1473-3099(14)70845-X PMC4178237

[180] Jeyanathan M, Fritz DK, Afkhami S, Aguirre E, Howie KJ, Zganiacz A, et al. Aerosol delivery, but not intramuscular injection, of adenovirus-vectored tuberculosis vaccine induces respiratory-mucosal immunity in humans. JCI Insight. 2022. 10.1172/jci.insight.155655. 34990408 10.1172/jci.insight.155655 PMC8855837

[181] Reyes-Sandoval A, Wyllie DH, Bauza K, Milicic A, Forbes EK, Rollier CS, Hill AV. CD8+ T effector memory cells protect against liver-stage malaria. J Immunol. 2011;187(3):1347–57. 10.4049/jimmunol.1100302. 21715686 10.4049/jimmunol.1100302 PMC4568294

[182] Sheerin D, Dold C, O’Connor D, Pollard AJ, Rollier CS. Distinct patterns of whole blood transcriptional responses are induced in mice following immunisation with adenoviral and poxviral vector vaccines encoding the same antigen. BMC Genomics. 2021;22(1): 777. 10.1186/s12864-021-08061-8. 34717548 10.1186/s12864-021-08061-8 PMC8556829

[183] Quinn KM, Zak DE, Costa A, Yamamoto A, Kastenmuller K, Hill BJ, et al. Antigen expression determines adenoviral vaccine potency independent of IFN and STING signaling. J Clin Investig. 2015;125(3):1129–46. 10.1172/Jci78280. 25642773 10.1172/JCI78280 PMC4362254

[184] Folegatti PM, Ewer KJ, Aley PK. Safety and immunogenicity of the ChAdOx1 nCoV-19 vaccine against SARS-CoV-2: a preliminary report of a phase 1/2, single-blind, randomised controlled trial. Lancet. 2020;396(10266):1884. 10.1016/S0140-6736(20)31604-4 PMC7445431 32702298

[185] Manjaly Thomas ZR, Satti I, Marshall JL, Harris SA, Lopez Ramon R, Hamidi A, et al. Alternate aerosol and systemic immunisation with a recombinant viral vector for tuberculosis, MVA85A: a phase I randomised controlled trial. PLoS Med. 2019;16(4): e1002790. 10.1371/journal.pmed.1002790. 31039172 10.1371/journal.pmed.1002790 PMC6490884

[186] Wu SP, Huang JY, Zhang Z, Wu JY, Zhang JL, Hu HN, et al. Safety, tolerability, and immunogenicity of an aerosolised adenovirus type-5 vector-based COVID-19 vaccine (Ad5-nCoV) in adults: preliminary report of an open-label and randomised phase 1 clinical trial. Lancet Infect Dis. 2021;21(12):1654–64. 10.1016/S1473-3099(21)00396-0. 34324836 10.1016/S1473-3099(21)00396-0 PMC8313090

[187] Alu A, Chen L, Lei H, Wei Y, Tian X, Wei X. Intranasal COVID-19 vaccines: from bench to bed. EBioMedicine. 2022;76: 103841. 10.1016/j.ebiom.2022.103841. 35085851 10.1016/j.ebiom.2022.103841 PMC8785603

[188] Morens DM, Taubenberger JK, Fauci AS. Rethinking next-generation vaccines for coronaviruses, influenzaviruses, and other respiratory viruses. Cell Host Microbe. 2023;31(1):146–57. 10.1016/j.chom.2022.11.016. 36634620 10.1016/j.chom.2022.11.016 PMC9832587

[189] Zhu F, Zhuang C, Chu K, Zhang L, Zhao H, Huang S, et al. Safety and immunogenicity of a live-attenuated influenza virus vector-based intranasal SARS-CoV-2 vaccine in adults: randomised, double-blind, placebo-controlled, phase 1 and 2 trials. Lancet Respir Med. 2022;10(8):749–60. 10.1016/S2213-2600(22)00131-X. 35644168 10.1016/S2213-2600(22)00131-X PMC9135375

[190] Sivanandam V, LaRocca CJ, Chen NHG, Fong YM, Warner SG. Oncolytic viruses and immune checkpoint inhibition: the best of both worlds. Mol Ther-Oncolytics. 2019;13:93–106. 10.1016/j.omto.2019.04.003. 31080879 10.1016/j.omto.2019.04.003 PMC6503136

[191] An D, Li K, Rowe DK, Diaz MCH, Griffin EF, Beavis AC et al. Protection of K18-hACE2 mice and ferrets against SARS-CoV-2 challenge by a single-dose mucosal immunization with a parainfluenza virus 5-based COVID-19 vaccine. Sci Adv. 2021;7(27). ARTN eabi5246. 10.1126/sciadv.abi5246. 10.1126/sciadv.abi5246 PMC11057785 34215591

[192] Ponce-de-León S, Torres M, Soto-Ramírez LE, Calva JJ, Santillán-Doherty P, Carranza-Salazar DE et al. Interim safety and immunogenicity results from an NDV-based COVID-19 vaccine phase I trial in Mexico. Npj Vaccines. 2023;8(1):67. 10.1038/s41541-023-00662-6. 37164959 10.1038/s41541-023-00662-6 PMC10170424

[193] Ku MW, Bourgine M, Authié P, Lopez J, Nemirov K, Moncoq F, et al. Intranasal vaccination with a lentiviral vector protects against SARS-CoV-2 in preclinical animal models. Cell Host Microbe. 2021;29(2):236. 10.1016/j.chom.2020.12.010. 33357418 10.1016/j.chom.2020.12.010 PMC7738935

[194] Vanaparthy R, Mohan G, Vasireddy D, Atluri P. Review of COVID-19 viral vector-based vaccines and COVID-19 variants. Infez Med. 2021;29(3):328–38. 10.53854/liim-2903-3. 35146337 10.53854/liim-2903-3 PMC8805485

[195] Rajanala K, Upadhyay AK. Vaccines for respiratory viruses-COVID and beyond. Vaccines. 2024;12(8): ARTN 936. 10.3390/vaccines12080936. 10.3390/vaccines12080936 PMC11360283 39204059

[196] Li J, Wellnitz S, Chi XS, Yue Y, Schmidt KA, Nguyen N, et al. Horizontal transmission of cytomegalovirus in a rhesus model despite high-level, vaccine-elicited neutralizing antibody and T-cell responses. J Infect Dis. 2022;226(4):585–94. 10.1093/infdis/jiac129. 35413121 10.1093/infdis/jiac129 PMC10147388

[197] van den Berg AIS, Yun CO, Schiffelers RM, Hennink WE. Polymeric delivery systems for nucleic acid therapeutics: Approaching the clinic. J Control Release. 2021;331:121–41. 10.1016/j.jconrel.2021.01.014. 33453339 10.1016/j.jconrel.2021.01.014

[198] Tan L, Sun X. Recent advances in mRNA vaccine delivery. Nano Res. 2018;11(10):5338–54. 10.1007/s12274-018-2091-z.

[199] Comes JDG, Pijlman GP, Hick TAH. Rise of the RNA machines - self-amplification in mRNA vaccine design. Trends Biotechnol. 2023;41(11):1417–29. 10.1016/j.tibtech.2023.05.007. 37328401 10.1016/j.tibtech.2023.05.007 PMC10266560

[200] Maruggi G, Zhang CL, Li JW, Ulmer JB, Yu D. mRNA as a Transformative Technology for Vaccine Development to Control Infectious Diseases. Mol Ther. 2019;27(4):757–72. 10.1016/j.ymthe.2019.01.020. 30803823 10.1016/j.ymthe.2019.01.020 PMC6453507

[201] Hajj KA, Whitehead KA. Tools for translation: non-viral materials for therapeutic mRNA delivery. Nat Rev Mater. 2017;2(10). ARTN 17056. 10.1038/natrevmats.2017.56.

[202] Linares-Fernandez S, Lacroix C, Exposito JY, Verrier B. Tailoring mRNA vaccine to balance innate/adaptive immune response. Trends Mol Med. 2020;26(3):311–23. 10.1016/j.molmed.2019.10.002. 31699497 10.1016/j.molmed.2019.10.002

[203] Gao YY, Guo Y. Research progress in the development of natural-product-based mucosal vaccine adjuvants. Frontiers in Immunology. 2023;14:1152855. 10.3389/fimmu.2023.1152855. 37090704 10.3389/fimmu.2023.1152855 PMC10113501

[204] van der Weken H, Cox E, Devriendt B. Advances in Oral Subunit Vaccine Design. Vaccines-Basel. 2021;9(1):1. 10.3390/vaccines9010001. 10.3390/vaccines9010001 PMC7822154 33375151

[205] Clements JD, Norton EB. The Mucosal Vaccine Adjuvant LT(R192G/L211A) or dmLT. Msphere. 2018;3(4):e00215–18. 10.1128/mSphere.00215-18. 30045966 10.1128/mSphere.00215-18 PMC6060342

[206] Lebens M, Terrinoni M, Karlsson SL, Larena M, Gustafsson-Hedberg T, Källgård S, et al. Construction and preclinical evaluation of mmCT, a novel mutant cholera toxin adjuvant that can be efficiently produced in genetically manipulated. Vaccine. 2016;34(18):2121–8. 10.1016/j.vaccine.2016.03.002. 26973069 10.1016/j.vaccine.2016.03.002

[207] Pan SC, Hsieh SM, Lin CF, Hsu YS, Chang M, Chang SC. A randomized, double-blind, controlled clinical trial to evaluate the safety and immunogenicity of an intranasally administered trivalent inactivated influenza vaccine with adjuvant LTh(alphaK): a phase I study. Vaccine. 2019;37(14):1994–2003. 10.1016/j.vaccine.2019.02.006. 30837170 10.1016/j.vaccine.2019.02.006

[208] Pan SC, Hsu WT, Lee WS, Wang NC, Chen TJ, Liu MC, et al. A double-blind, randomized controlled trial to evaluate the safety and immunogenicity of an intranasally administered trivalent inactivated influenza vaccine with the adjuvant LTh(αK): A phase II study. Vaccine. 2020;38(5):1048–56. 10.1016/j.vaccine.2019.11.047. 31812463 10.1016/j.vaccine.2019.11.047

[209] Miquel-Clopés A, Bentley EG, Stewart JP, Carding SR. Mucosal vaccines and technology. Clin Exp Immunol. 2019;196(2):205–14. 10.1111/cei.13285. 30963541 10.1111/cei.13285 PMC6468177

[210] Lycke N. Recent progress in mucosal vaccine development: potential and limitations. Nat Rev Immunol. 2012;12(8):592–605. 10.1038/nri3251. 22828912 10.1038/nri3251

[211] Zhao BL, Yang JY, He B, Li X, Yan H, Liu SN, et al. A safe and effective mucosal RSV vaccine in mice consisting of RSV phosphoprotein and flagellin variant. Cell Rep. 2021;36(3): 109401. 10.1016/j.celrep.2021.109401. 34289371 10.1016/j.celrep.2021.109401

[212] Yang JY, Liu MQ, Liu L, Li X, Xu MX, Lin HF, et al. A triple-RBD-based mucosal vaccine provides broad protection against SARS-CoV-2 variants of concern. Cell Mol Immunol. 2022;19(11):1279–89. 10.1038/s41423-022-00929-3. 36220993 10.1038/s41423-022-00929-3 PMC9552159

[213] Song L, Xiong D, Song HQ, Wu LL, Zhang MH, Kang XL et al. Mucosal and Systemic immune responses to influenza H7n9 Antigen HA1–2Co-Delivered intranasally With Flagellin or Polyethyleneimine in Mice and Chickens (vol 8, pg 326, 2017). Frontiers in Immunology. 2018;9. ARTN 1846. 10.3389/fimmu.2018.01846. 10.3389/fimmu.2017.00326 PMC5380672 28424686

[214] Baldridge JR, Yorgensen Y, Ward JR, Ulrich JT. Monophosphoryl lipid a enhances mucosal and systemic immunity to vaccine antigens following intranasal administration. Vaccine. 2000;18(22):2416–25. 10.1016/S0264-410x(99)00572-1. 10738099 10.1016/s0264-410x(99)00572-1

[215] Alu A, Chen L, Lei H, Wei YQ, Tian XH, Wei XW. Intranasal COVID-19 vaccines: From bench to bed. Ebiomedicine. 2022;76:103841. 10.1016/j.ebiom.2022.103841. 35085851 10.1016/j.ebiom.2022.103841 PMC8785603

[216] Sabbaghi A, Malek M, Abdolahi S, Miri SM, Alizadeh L, Samadi M et al. A formulated poly (I:C)/CCL21 as an effective mucosal adjuvant for gamma-irradiated influenza vaccine. Virol J. 2021;18(1):201-215. 10.1186/s12985-021-01684-z. 34627297 10.1186/s12985-021-01672-3 PMC8501930

[217] McNally B, Willette M, Ye F, Partida-Sanchez S, Flaño E. Intranasal administration of dsRNA analog poly(I:C) induces interferon-α receptor-dependent accumulation of antigen experienced T cells in the airways. PLoS One. 2012;7(12): ARTN e51351. 10.1371/journal.pone.0051351. 10.1371/journal.pone.0051351 PMC3517467 23236482

[218] Hong SH, Byun YH, Nguyen CT, Kim SY, Seong BL, Park S, et al. Intranasal administration of a flagellin-adjuvanted inactivated influenza vaccine enhances mucosal immune responses to protect mice against lethal infection. Vaccine. 2012;30(2):466–74. 10.1016/j.vaccine.2011.10.058. 22051136 10.1016/j.vaccine.2011.10.058

[219] Hjelm BE, Kilbourne J, Herbst-Kralovetz MM. TLR7 and 9 agonists are highly effective mucosal adjuvants for norovirus virus-like particle vaccines. Hum Vacc Immunother. 2014;10(2):410–6. 10.4161/hv.27147. 10.4161/hv.27147 PMC4185889 24280723

[220] Rhee JH, Lee SE, Kim SY. Mucosal vaccine adjuvants update. Clin Exp Vaccine Res. 2012;1(1):50–63. 10.7774/cevr.2012.1.1.50. 23596577 10.7774/cevr.2012.1.1.50 PMC3623511

[221] Kayamuro H, Yoshioka Y, Abe Y, Arita S, Katayama K, Nomura T, et al. Interleukin-1 family cytokines as mucosal vaccine adjuvants for induction of protective immunity against influenza virus. J Virol. 2010;84(24):12703–12. 10.1128/Jvi.01182-10. 20881038 10.1128/JVI.01182-10 PMC3004317

[222] Sun HX, Xie Y, Ye YP. Advances in saponin-based adjuvants. Vaccine. 2009;27(12):1787–96. 10.1016/j.vaccine.2009.01.091. 19208455 10.1016/j.vaccine.2009.01.091

[223] Swarnalekha N, Schreiner D, Litzler LC, Iftikhar S, Kirchmeier D, Künzli M et al. T resident helper cells promote humoral responses in the lung. Sci Immunol. 2021;6(55):eabb6808. 10.1126/sciimmunol.abb6808. 33419790 10.1126/sciimmunol.abb6808 PMC8063390

[224] Singh S, Nehete PN, Yang GJ, He H, Nehete B, Barry MA, Sastry KJ. Enhancement of mucosal immunogenicity of protein and viral vectored vaccines by the Nkt cell agonist alpha-galactosylceramide as adjuvant. J Med Primatol. 2015;44(5):338. 10.3390/vaccines2040686 PMC4278383 25553254

[225] Lee YS, Lee KA, Lee JY, Kang MH, Song YC, Baek DJ, et al. An α-GalCer analogue with branched acyl chain enhances protective immune responses in a nasal influenza vaccine. Vaccine. 2011;29(3):417–25. 10.1016/j.vaccine.2010.11.005. 21087689 10.1016/j.vaccine.2010.11.005

[226] Zhou G, Hollenberg MD, Vliagoftis H, Kane KP. Protease-activated receptor 2 agonist as adjuvant: augmenting development of protective memory CD8 T cell responses induced by influenza virosomes. J Immunol. 2019;203(2):441–52. 10.4049/jimmunol.1800915. 31182479 10.4049/jimmunol.1800915

[227] Shim S, Yoo HS. The application of mucoadhesive chitosan nanoparticles in nasal drug delivery. Mar Drugs. 2020;18(12): ARTN 605. 10.3390/md18120605. 10.3390/md18120605 PMC7759871 33260406

[228] Wegmann F, Gartlan KH, Harandi AM, Brinckmann SA, Coccia M, Hillson WR, et al. Polyethyleneimine is a potent mucosal adjuvant for viral glycoprotein antigens. Nat Biotechnol. 2012;30(9):883. 10.1038/nbt.2344. 22922673 10.1038/nbt.2344 PMC3496939

[229] Lei H, Alu A, Yang JY, He C, Hong WQ, Cheng ZS et al. Cationic nanocarriers as potent adjuvants for recombinant S-RBD vaccine of SARS-CoV-2. Signal Transduct Tar. 2020;5(1):291. 10.1038/s41392-020-00434-x. 10.1038/s41392-020-00434-x PMC7729145 33311439

[230] Lei H, Alu A, Yang JY, He X, He C, Ren WY et al. Cationic crosslinked carbon dots-adjuvanted intranasal vaccine induces protective immunity against Omicron-included SARS-CoV-2 variants. Nat Commun. 2023;14(1):2678. 10.1038/s41467-023-38066-8. 37160882 10.1038/s41467-023-38066-8 PMC10169129

[231] Ren ST, Zhang XM, Sun PF, Sun LJ, Guo X, Tian T, et al. Intranasal immunization using Mannatide as a novel adjuvant for an inactivated influenza vaccine and its adjuvant effect compared with MF59. PLoS One. 2017;12(1):e0169501. 10.1371/journal.pone.0169501. 28052136 10.1371/journal.pone.0169501 PMC5215226

[232] Lei H, Hong W, Yang J, He C, Zhou Y, Zhang Y et al. Intranasal delivery of a subunit protein vaccine provides protective immunity against JN.1 and XBB-lineage variants. Signal Transduct Target Ther. 2024;9(1):311. 10.1038/s41392-024-02025-6. 39562542 10.1038/s41392-024-02025-6 PMC11577066

[233] Du Y, Xu Y, Feng J, Hu L, Zhang Y, Zhang B, et al. Intranasal administration of a recombinant RBD vaccine induced protective immunity against SARS-CoV-2 in mouse. Vaccine. 2021;39(16):2280–7. 10.1016/j.vaccine.2021.03.006. 33731271 10.1016/j.vaccine.2021.03.006 PMC7934688

[234] Bai ZY, Wan DD, Lan TX, Hong WQ, Dong HH, Wei YQ, Wei XW. Nanoplatform based intranasal vaccines: current progress and clinical challenges. ACS Nano. 2024;18(36):24650–81. 10.1021/acsnano.3c10797. 39185745 10.1021/acsnano.3c10797 PMC11394369

[235] Juan A, Cimas FJ, Bravo I, Pandiella A, Ocaña A, Alonso-Moreno C. An overview of antibody conjugated polymeric nanoparticles for breast cancer therapy. Pharmaceutics. 2020;12(9): ARTN 802. 10.3390/pharmaceutics12090802. 10.3390/pharmaceutics12090802 PMC7558516 32854255

[236] Ye QN, Wang Y, Shen S, Xu CF, Wang J. Biomaterials-Based Delivery of Therapeutic Antibodies for Cancer Therapy. Adv Healthc Mater. 2021;10(11):2002139. 10.1002/adhm.202002139. 10.1002/adhm.202002139 33870637

[237] Lee JW, Khang D. Mucosal delivery of nanovaccine strategy against COVID-19 and its variants. Acta Pharm Sin B. 2023;13(7):2897–925. 10.1016/j.apsb.2022.11.022. 10.1016/j.apsb.2022.11.022 PMC9676163 36438851

[238] Dhillon GS, Kaur S, Brar SK, Verma M. Green synthesis approach: extraction of chitosan from fungus mycelia. Crit Rev Biotechnol. 2013;33(4):379–403. 10.3109/07388551.2012.717217. 23078670 10.3109/07388551.2012.717217

[239] Al-Nemrawi N, Nimrawi S. A novel formulation of chitosan nanoparticles functionalized with titanium dioxide nanoparticles. J Adv Pharm Technol Res. 2021;12(4):402–7. 10.4103/japtr.japtr_22_21. 34820317 10.4103/japtr.japtr_22_21 PMC8588920

[240] Wu D, Zhu L, Li Y, Zhang X, Xu S, Yang G, Delair T. Chitosan-based colloidal polyelectrolyte complexes for drug delivery: a review. Carbohydr Polym. 2020;238: 116126. 10.1016/j.carbpol.2020.116126. 32299572 10.1016/j.carbpol.2020.116126

[241] Pawar D, Mangal S, Goswami R, Jaganathan KS. Development and characterization of surface modified PLGA nanoparticles for nasal vaccine delivery: effect of mucoadhesive coating on antigen uptake and immune adjuvant activity. Eur J Pharm Biopharm. 2013;85(3 Pt A):550–9. 10.1016/j.ejpb.2013.06.017. 23831265 10.1016/j.ejpb.2013.06.017

[242] Mignani S, Shi XY, Karpus A, Lentini G, Majoral JP. Functionalized dendrimer platforms as a new forefront arsenal targeting SARS-CoV-2: an opportunity. Pharmaceutics. 2021;13(9): ARTN 1513. 10.3390/pharmaceutics13091513. 10.3390/pharmaceutics13091513 PMC8466088 34575589

[243] Zaman M, Chandrudu S, Toth I. Strategies for intranasal delivery of vaccines. Drug Deliv Transl Res. 2013;3(1):100–9. 10.1007/s13346-012-0085-z. 23316448 10.1007/s13346-012-0085-z PMC3539070

[244] Eygeris Y, Patel S, Jozic A, Sahay G. Deconvoluting lipid nanoparticle structure for messenger RNA delivery. Nano Lett. 2020;20(6):4543–9. 10.1021/acs.nanolett.0c01386. 32375002 10.1021/acs.nanolett.0c01386

[245] Giddam AK, Zaman M, Skwarczynski M, Toth I. Liposome-based delivery system for vaccine candidates: constructing an effective formulation (vol 7, pg 1877, 2012). Nanomedicine-Uk. 2013;8(2):312-. 10.2217/nnm.12.157 23249332

[246] Nordly P, Madsen HB, Nielsen HM, Foged C. Status and future prospects of lipid-based particulate delivery systems as vaccine adjuvants and their combination with immunostimulators. Expert Opin Drug Deliv. 2009;6(7):657–72. 10.1517/17425240903018863. 19538037 10.1517/17425240903018863

[247] Sapra P, Allen TM. Ligand-targeted liposomal anticancer drugs. Prog Lipid Res. 2003;42(5):439–62. 10.1016/S0163-7827(03)00032-8. 12814645 10.1016/s0163-7827(03)00032-8

[248] Fan Y, Sahdev P, Ochyl LJ, Akerberg J, Moon JJ. Cationic liposome-hyaluronic acid hybrid nanoparticles for intranasal vaccination with subunit antigens. J Control Release. 2015;208:121–9. 10.1016/j.jconrel.2015.04.010. 25869965 10.1016/j.jconrel.2015.04.010 PMC4430437

[249] Hartwell BL, Melo MB, Xiao P, Lemnios AA, Li N, Chang JYH et al. Intranasal vaccination with lipid-conjugated immunogens promotes antigen transmucosal uptake to drive mucosal and systemic immunity. Sci Transl Med. 2022;14(654):eabn1413. 10.1126/scitranslmed.abn1413. 10.1126/scitranslmed.abn1413 PMC9835395 35857825

[250] Tada R, Suzuki H, Takahashi S, Negishi Y, Kiyono H, Kunisawa J, Aramaki Y. Nasal vaccination with pneumococcal surface protein A in combination with cationic liposomes consisting of DOTAP and DC-chol confers antigen-mediated protective immunity against Streptococcus pneumoniae infections in mice. Int Immunopharmacol. 2018;61:385–93. 10.1016/j.intimp.2018.06.027. 29945026 10.1016/j.intimp.2018.06.027

[251] Amacker M, Engler O, Kammer AR, Vadrucci S, Oberholzer D, Cerny A, Zurbriggen R. Peptide-loaded chimeric influenza virosomes for efficient in vivo induction of cytotoxic T cells. Int Immunol. 2005;17(6):695–704. 10.1093/intimm/dxh249. 15843436 10.1093/intimm/dxh249

[252] Keikha R, Daliri K, Jebali A. The use of nanobiotechnology in immunology and vaccination. Vaccines. 2021. 10.3390/vaccines9020074. 33494441 10.3390/vaccines9020074 PMC7910821

[253] Syomin BV, Ilyin YV. Virus-like particles as an instrument of vaccine production. Molecular Biology. 2019;53(3):323–34. 10.1134/S0026893319030154. 32214478 10.1134/S0026893319030154 PMC7088979

[254] Rothen DA, Krenger PS, Nonic A, Balke I, Vogt AS, Chang X, et al. Intranasal administration of a virus like particles-based vaccine induces neutralizing antibodies against SARS-CoV-2 and variants of concern. Allergy. 2022;77(8):2446–58. 10.1111/all.15311. 35403221 10.1111/all.15311 PMC9111403

[255] Samrat SK, Tharappel AM, Li Z, Li H. Prospect of SARS-CoV-2 spike protein: potential role in vaccine and therapeutic development. Virus Res. 2020;288: 198141. 10.1016/j.virusres.2020.198141. 32846196 10.1016/j.virusres.2020.198141 PMC7443330

[256] Ali H, Akbar M, Iqbal B, Ali F, Sharma NK, Kumar N, et al. Virosome: An engineered virus for vaccine delivery. Saudi Pharm J. 2023;31(5):752–64. 10.1016/j.jsps.2023.03.016. 37181145 10.1016/j.jsps.2023.03.016 PMC10172599

[257] Watanabe K. Bacterial membrane vesicles (MVs): novel tools as nature- and nano-carriers for immunogenic antigen, enzyme support, and drug delivery. Appl Microbiol Biot. 2016;100(23):9837–43. 10.1007/s00253-016-7916-7. 10.1007/s00253-016-7916-7 27761637

[258] Schulz E, Goes A, Garcia R, Panter F, Koch M, Müller R, et al. Biocompatible bacteria-derived vesicles show inherent antimicrobial activity. J Control Release. 2018;290:46–55. 10.1016/j.jconrel.2018.09.030. 30292423 10.1016/j.jconrel.2018.09.030

[259] Thapa HB, Muller AM, Camilli A, Schild S. An intranasal vaccine based on outer membrane vesicles against SARS-CoV-2. Front Microbiol. 2021;12: 752739. 10.3389/fmicb.2021.752739. 34803974 10.3389/fmicb.2021.752739 PMC8602898

[260] Aznar MA, Tinari N, Rullan AJ, Sanchez-Paulete AR, Rodriguez-Ruiz ME, Melero I. Intratumoral delivery of immunotherapy-act locally. Think globally. J Immunol. 2017;198(1):31–9. 10.4049/jimmunol.1601145. 27994166 10.4049/jimmunol.1601145

[261] Pritsch M, Ben-Khaled N, Chaloupka M, Kobold S, Berens-Riha N, Peter A et al. Comparison of Intranasal Outer Membrane Vesicles with Cholera Toxin and Injected MF59C.1 as Adjuvants for Malaria Transmission Blocking Antigens AnAPN1 and Pfs48/45. J Immunol Res. 2016;2016:3576028. 10.1155/2016/3576028. 10.1155/2016/3576028 PMC4863099 27239480

[262] van der Ley PA, Zariri A, van Riet E, Oosterhoff D, Kruiswijk CP. An Intranasal OMV-Based Vaccine Induces High Mucosal and Systemic Protecting Immunity Against a SARS-CoV-2 Infection. Frontiers in Immunology. 2021;12:781280. 10.3389/fimmu.2021.781280. 10.3389/fimmu.2021.781280 PMC8721663 34987509

[263] Crothers JW, Norton EB. Recent advances in enterotoxin vaccine adjuvants. Curr Opin Immunol. 2023;85: 102398. 10.1016/j.coi.2023.102398. 37976963 10.1016/j.coi.2023.102398 PMC11258862

[264] Pizza M, Giuliani MM, Fontana MR, Monaci E, Douce G, Dougan G, et al. Mucosal vaccines: non toxic derivatives of LT and CT as mucosal adjuvants. Vaccine. 2001;19(17–19):2534–41. 10.1016/S0264-410x(00)00553-3. 11257389 10.1016/s0264-410x(00)00553-3

[265] Banerjee S, Medina-Fatimi A, Nichols R, Tendler D, Michetti M, Simon J, et al. Safety and efficacy of low dose Escherichia coli enterotoxin adjuvant for urease based oral immunisation against Helicobacter pylori in healthy volunteers. Gut. 2002;51(5):634–40. 10.1136/gut.51.5.634. 12377799 10.1136/gut.51.5.634 PMC1773429

[266] Cheng Q, Yang Y, Gao JQ. Infectivity of human coronavirus in the brain. Ebiomedicine. 2020;56:102799. 10.1016/j.ebiom.2020.102799. 32474399 10.1016/j.ebiom.2020.102799 PMC7255711

[267] Huang JH, Zheng MJ, Tang X, Chen YX, Tong AP, Zhou LX. Potential of SARS-CoV-2 to Cause CNS Infection: Biologic Fundamental and Clinical Experience. Front Neurol. 2020;11:659. 10.3389/fneur.2020.00659. 32625165 10.3389/fneur.2020.00659 PMC7314941

[268] Pavot V, Rochereau N, Genin C, Verrier B, Paul S. New insights in mucosal vaccine development. Vaccine. 2012;30(2):142–54. 10.1016/j.vaccine.2011.11.003. 22085556 10.1016/j.vaccine.2011.11.003

[269] Squier CA, Mantz MJ, Schilievert PM, Davis CC. Porcine vagina as a model for studying permeability and pathogenesis in mucosa. J Pharm Sci-Us. 2008;97(1):9–21. 10.1002/jps.21077. 10.1002/jps.21077 17721937

[270] Kawai T, Akira S. The role of pattern-recognition receptors in innate immunity: update on Toll-like receptors. Nat Immunol. 2010;11(5):373–84. 10.1038/ni.1863. 20404851 10.1038/ni.1863

[271] van Egmond M, Damen CA, van Spriel AB, Vidarsson G, van Garderen E, van de Winkel JG. IgA and the IgA Fc receptor. Trends Immunol. 2001;22(4):205–11. 10.1016/s1471-4906(01)01873-7. 11274926 10.1016/s1471-4906(01)01873-7

[272] Bruder MC, Spanhaak S, Bruijntjes JP, Michielsen CP, Vos JG, Kuper CF. Intestinal T lymphocytes of different rat strains in immunotoxicity. Toxicol Pathol. 1999;27(2):171–9. 10.1177/019262339902700204. 10207981 10.1177/019262339902700204

[273] de Silva TI, Gould V, Mohammed NI, Cope A, Meijer A, Zutt I, et al. Comparison of mucosal lining fluid sampling methods and influenza-specific IgA detection assays for use in human studies of influenza immunity. J Immunol Methods. 2017;449:1–6. 10.1016/j.jim.2017.06.008. 28647455 10.1016/j.jim.2017.06.008 PMC7619426

[274] Jochems SP, Piddock K, Rylance J, Adler H, Carniel BF, Collins A, et al. Novel analysis of immune cells from nasal microbiopsy demonstrates reliable, reproducible data for immune populations, and superior cytokine detection compared to nasal wash. PLoS One. 2017;12(1): e0169805. 10.1371/journal.pone.0169805. 28107457 10.1371/journal.pone.0169805 PMC5249128

[275] Nizard M, Roussel H, Diniz MO, Karaki S, Tran T, Voron T et al. Induction of resident memory T cells enhances the efficacy of cancer vaccine. Nat Commun. 2017;8:15221. 10.1038/ncomms15221. 28537262 10.1038/ncomms15221 PMC5458068

[276] Blanc C, Hans S, Tran T, Granier C, Saldman A, Anson M et al. Targeting Resident Memory T Cells for Cancer Immunotherapy. Front Immunol. 2018;9:1722. 10.3389/fimmu.2018.01722. 10.3389/fimmu.2018.01722 PMC6072845 30100906

[277] Parkhurst MR, Robbins PF, Tran E, Prickett TD, Gartner JJ, Jia L, et al. Unique neoantigens arise from somatic mutations in patients with gastrointestinal cancers. Cancer Discov. 2019;9(8):1022–35. 10.1158/2159-8290.Cd-18-1494. 31164343 10.1158/2159-8290.CD-18-1494 PMC7138461

[278] Jiang T, Cheng RR, Pan YW, Zhang HH, He Y, Su CX et al. Heterogeneity of neoantigen landscape between primary lesions and their matched metastases in lung cancer. Transl Lung Cancer R. 2020;9(2):246. 10.21037/tlcr.2020.03.03. 10.21037/tlcr.2020.03.03 PMC7225166 32420064

[279] Sahin U, Derhovanessian E, Miller M, Kloke BP, Simon P, Löwer M, et al. Personalized RNA mutanome vaccines mobilize poly-specific therapeutic immunity against cancer. Nature. 2017;547(7662):222. 10.1038/nature23003. 28678784 10.1038/nature23003

[280] Ott PA, Hu ZT, Keskin DB, Shukla SA, Sun J, Bozym DJ et al. An immunogenic personal neoantigen vaccine for patients with melanoma. Nature. 2018;555(7696):217-402. 10.1038/nature25145. 10.1038/nature25145 PMC6064631 29542692

[281] Nakamura Y, Kimura S, Hase K. M cell-dependent antigen uptake on follicle-associated epithelium for mucosal immune surveillance. Inflamm Regen. 2018;38:15. 10.1186/s41232-018-0072-y. 30186536 10.1186/s41232-018-0072-y PMC6120081

[282] Li Y, Jin L, Chen TX. The Effects of Secretory IgA in the Mucosal Immune System. Biomed Res Int. 2020;2020:2032057. 10.1155/2020/2032057. 31998782 10.1155/2020/2032057 PMC6970489

[283] Al-Talib M, Dimonte S, Humphreys IR. Mucosal T-cell responses to chronic viral infections: implications for vaccine design. Cell Mol Immunol. 2024;21(9):982–98. 10.1038/s41423-024-01140-2. 38459243 10.1038/s41423-024-01140-2 PMC11364786

[284] Pollard AJ, Bijker EM. A guide to vaccinology: from basic principles to new developments Nature Reviews Immunology (Dec, 10.1038/s41577-020-00479-7, 2020). 2021;21(2):129. . 10.1038/s41577-020-00497-5 PMC8095270 33402728

